# Effects of Different Drying Methods on Amino Acid Metabolite Content and Quality of *Ophiocordyceps sinensis* by LC-MS/MS Combined with Multivariate Statistical Methods

**DOI:** 10.3390/metabo14080459

**Published:** 2024-08-18

**Authors:** Mengjun Xiao, Tao Wang, Chuyu Tang, Min He, Yuling Li, Xiuzhang Li

**Affiliations:** State Key Laboratory of Plateau Ecology and Agriculture, Qinghai Academy of Animal and Veterinary Science, Qinghai University, Xining 810016, China; ys220951310549@qhu.edu.cn (M.X.); yb1909000079@qhu.edu.cn (T.W.); yb230909000082@qhu.edu.cn (C.T.); ys220951310541@qhu.edu.cn (M.H.)

**Keywords:** *Ophiocordyceps sinensis*, amino acid, LC-MS/MS, drying method, principal component analysis

## Abstract

*Ophiocordyceps sinensis*, a medicinal fungus utilized in traditional Chinese medicine, exhibits a range of biological activities and pharmacological functions. In this study, we determined the amino acid composition of 94 amino acids in *Ophiocordyceps sinensis* using liquid chromatography–tandem mass spectrometry (LC-MS/MS). Fresh samples of *Ophiocordyceps sinensis* were analyzed under three different drying methods: vacuum freeze drying (DG), oven drying (HG), and air drying (YG). This investigation aims to assess the effects of these drying methods on the content and quality of amino acid metabolites in *Ophiocordyceps sinensis*. Principal component analysis (PCA) and hierarchical cluster analysis (HCA) were employed for sample classification and the identification of differentially accumulated metabolites (DAMs). The results revealed the detection of 79 amino acid metabolites, which included elevated levels of oxidized L-glutamic acid, L-glutamic acid, and glutathione. Differential amino acid metabolites that met the criteria of fold change (|FC|) ≥ 2, *p*-value (*p*) ≤ 0.5, and variable importance in projection (VIP) ≥ 1 were analyzed. Significant differences in 48 amino acid metabolites between the groups were primarily related to protein synthesis. According to the KEGG analysis, all three comparison samples exhibited significant enrichment in several pathways. These pathways included the interaction of neuroactive ligands with receptors, the metabolism of cysteine and methionine, and the biosynthesis of plant hormones. The variations in amino acid metabolite levels observed across the three drying methods may be attributed to the degradation of proteins or amino acid metabolites, influenced by several factors, including temperature, enzyme activity, and moisture content. Additionally, Maillard and oxidative reactions involving substances such as amino acids, sugars, and oxygen may also play a significant role. This study demonstrates that various drying methods significantly influence the amino acid metabolite content of *Ophiocordyceps sinensis*. Therefore, the selection of drying methods should be tailored to meet specific requirements. This research provides important insights into the metabolite composition of *Ophiocordyceps sinensis* under different drying techniques, thereby contributing to a more comprehensive understanding of its nutritional and therapeutic properties.

## 1. Introduction

*Ophiocordyceps sinensis*, a valuable tonic herb in China, possesses a nutrient profile that exceeds that of ginseng. It serves both as a medicinal substance and a delicacy, rendering it a highly sought-after food item. This fungus is predominantly found in high-altitude regions, specifically between 3500 and 5000 m above sea level [1,2]. It has a faintly fishy odor and a bitter taste and is recognized for its properties that tonify the kidneys, benefit the lungs, halt bleeding, and resolve phlegm. In China, it is regarded as one of the three principal tonic herbs, alongside ginseng and deer antler. As a traditional Chinese medicine, *O. sinensis* has a wide range of medicinal values. It is commonly used in the treatment of hyperglycemia, renal failure and respiratory disorders [3,4]. Due to its unique flavor and rich array of active ingredients, including polysaccharides, amino acids, and nucleosides, *O. sinensis* has gained popularity as one of the most sought-after tonic herbs globally [5,6,7,8].

Fresh *O. sinensis* is characterized by its high water content, which makes it highly susceptible to deterioration and results in a short shelf-life. Although fresh *O. sinensis* possesses superior qualities and nutritional values, including exceptional bioactivities, compared to dried *O. sinensis*, drying remains the predominant method for its preservation [9,10]. Furthermore, drying is essential for food preservation, as it enables cost-effective storage and transportation by reducing product weight, thereby enhancing the potential for distribution to various locations [11,12]. Additionally, drying plays a crucial role in food preservation by facilitating cost-effective storage and transportation through the reduction of product weight, thereby improving distribution potential to various locations [13,14,15].

In recent years, drying techniques have become a widely employed method for the preservation of food and pharmaceuticals. A significant body of research has focused on the impact of drying conditions and efficiency on product quality. For instance, one study utilized microwave-assisted pulse jet bed freeze drying (MPSFD) to examine its effects on the volatile compounds and structural characteristics of *O. sinensis* [16]. Another investigation analyzed the influence of spray-drying process parameters on the production of herbal powder intended for medicinal purposes [17]. This research offered valuable insights into the relationship between process variables, the characteristics of the final product, and overall production efficiency. Furthermore, studies have assessed the effects of various drying methods, including hot air drying (HAD), vacuum freeze drying (VFD), vacuum drying (VD), and intermittent microwave hot air drying (MW-HAD), on the taste and flavor profile of *Cordyceps militaris* [18]. The findings provide crucial information regarding the development of flavors in *Cordyceps militaris* following the drying process.

*O. sinensis* exhibits distinct characteristics in terms of appearance, flavor, and aroma [19,20]. Metabolites play a crucial role in contributing to the nutrients, taste, and flavor of *O. sinensis*. Amino acids, which serve as precursors to volatile compounds, are closely associated with the quality traits of *O. sinensis* [21,22]. As fundamental building blocks of proteins, these amino acids can form peptide chains through dehydration. A deficiency in amino acids can impede protein synthesis and organism development, leading to symptoms such as memory loss, weakness, and swelling [23,24]. In plants, a deficiency of amino acids can result in root decay and stunted growth, while fungi lacking amino acids may experience metabolic imbalances [25]. Amino acids, as active and nutritional components of *O. sinensis* protein hydrolysate, have been shown to enhance immunity, among other benefits. Previous studies indicate that nucleosides and amino acids isolated from *O. sinensis* can provide protection against cyclophosphamide-induced myelosuppression in mice [26].

There are limited studies examining the mechanisms by which different drying methods affect the amino acid metabolites of *O. sinensis.* The content of amino acid metabolites in *O. sinensis* may vary depending on the drying methods employed, which could subsequently influence the levels of essential and flavorful amino acids through distinct metabolic pathways. It is essential to investigate how different drying methods impact the metabolic characteristics of *O. sinensis* to advance knowledge in this area. 

In this study, 94 amino acids were quantified by liquid chromatography–tandem mass spectrometry (LC-MS/MS) platform and fresh *O. sinensis* was used as an experimental control to clarify the effects of three different drying methods under (DG, HG and YG) on fresh *O. sinensis.* The primary objectives were to enhance understanding of the varying responses of amino acid metabolites to different drying methods, identify biomarkers that exhibit significant differences, and provide a comprehensive overview of the amino acid metabolite expression profiles of *O. sinensis* under various drying techniques.

## 2. Materials and Procedures

### 2.1. Source of Materials

High-performance liquid chromatography-grade acetonitrile and methanol were procured from Merck, located in Darmstadt, Germany. MilliQ water, used in the experimental setup, was supplied by Millipore in Bradford, USA. The standard was provided by Sigma-Aldrich in St. Louis, MO, USA, with ammonium acetate and formic acid also obtained from the same supplier. Details of the 94 amino acid standards (Table S1) are stored at −20 °C. All samples were purchased from Qinghai Baohuitang Biotechnology Co., based in Xining City, Qinghai Province, China (CK, DG, HG, and YG; 3 repetitions). The purchased sample included a total of 12 g of fresh *O. sinensis* (CK), collected in June 2023 from Maqin County (96°39′13″ E, 34°7′16″ N, H: 4113), Guoluo Tibetan Autonomous Prefecture, Qinghai Province, China. For this experiment, intact specimens were collected from the soil, placed in liquid nitrogen immediately after leaving the soil, and kept in an ultra-low-temperature chamber at −80 °C (Haier Corporation, Qingdao, China).

Three distinct drying methods—vacuum freeze drying (DG), oven drying (HG), and air drying (YG)—were employed to dry three grams of the whole herb, respectively. The drying process continued until a constant weight was attained. In the DG method, fresh *O. sinensis* was arranged in a single layer on trays to prevent overlapping and was subsequently freeze-dried using a vacuum freeze-dryer at −35 °C. This process included a pre-cooling phase lasting 3 h, followed by 25 h of low-temperature, high-vacuum freeze drying until a stable weight was achieved. In contrast, the HG method involved uniformly placing fresh *O. sinensis* on trays within an electrically heated thermostatic drying oven set at 60 °C, using hot air until a consistent weight was reached, at which point the samples were removed. For the YG process, fresh *O. sinensis* was laid flat in a cool, dry environment with controlled ambient conditions, specifically a temperature range of 20–25 °C and relative humidity of 50–65%. This setup was maintained until the specimen reached a stable weight. Subsequently, the moisture content was determined using the gravimetric method.

### 2.2. Metabolite Extraction

Place 50 milligrams (±2.5 mg) of the *O. sinensis* sample into a 2 mL tube. Subsequently, add 500 μL of pre-cooled 70% methanol aqueous solution from Merck KGaA, Darmstadt, Germany, maintained at −20 °C. Shake the mixture for 3 min, followed by centrifugation for 10 min at 4 °C and 12,000 rpm. Extract 300 μL of the supernatant into a 1.5 mL tube and allow it to rest for 30 min at −20 °C.

### 2.3. Analysis Using LC-MS/MS

The LC-MS/MS investigation was conducted using well-established methodologies [27,28]. An UltraPerformance Liquid Chromatography (UPLC) ExionLC™ AD (https://sciex.com.cn/, accessed on 3 April 2024) and Tandem Mass Spectrometry (MS/MS) QTRAP^®^ 6500+ (https://sciex.com.cn/) systems were employed to analyze the samples. The chromatographic columns utilized were ACQUITY BEH Amide columns (1.7 µm, 100 mm × 2.1 mm i.d.). The lower limit of detection (LOD) was determined to be 0.05 ng/mL. The column temperature, injection volume (LOQ), and flow rate were set to 40 °C, 2 µL, and 0.4 mL/min, respectively. The mobile phase consisted of two components: phase A contained ultrapure water with 2 mM ammonium acetate and 0.04% formic acid, while phase B comprised acetonitrile with 2 mM ammonium acetate and 0.04% formic acid. The gradient elution process commenced with a 10:90 (*v*/*v*) ratio of A/B from 0 to 1.2 min, transitioned to a 40:60 (*v*/*v*) ratio over a period of 9 min, further adjusted to a 60:40 (*v*/*v*) ratio between the 10th and 11th minutes, and ultimately reverted to a 10:90 (*v*/*v*) ratio from 11.01 to 15 min. The mass spectrometry conditions were as follows: the electrospray ionization (ESI) temperature was set at 550 °C, with a mass spectrometry voltage of 5500 V in positive ion mode and −4500 V in negative ion mode. The Curtain Gas (CUR) was maintained at 35 psi. In the Q-Trap 6500+, each ion pair was scanned and detected based on an optimized declustering potential (DP) and collision energy (CE). Quantification was achieved using triple quadrupole mass spectrometry in Multiple Reaction Monitoring (MRM) mode. Following the acquisition of mass spectrometry data from various samples, the chromatographic peaks of all targets were integrated and quantitatively analyzed using a standard curve. A Metware V 1.0 Database (MWDB) was established from the standards to facilitate the characterization of the mass spectrometry data. Aliquots of mixed *O. sinensis* samples were combined to produce quality control (QC) samples to ensure instrumental consistency. They were introduced after every 10 analytical samples throughout the analysis. The standard deviation (SD) and relative standard deviation (RSD) values of the assay results for the three post-extraction samples corresponding to each sample are presented in Table S2.

### 2.4. Data Processing

LC-MS/MS raw data were imported into the MultiQuant 3.0.3 application for data processing. Data matrices for retention time, mass-to-charge ratio, and peak intensity were generated by excluding characteristic peaks with a relative standard deviation (RSD) greater than 30% in the QC samples. In LC-MS analysis, the retention times of metabolites, along with the precursor and fragment ions in Multiple Reaction Monitoring (MRM) mode, were determined from metabolite standards, facilitating the identification and quantification of metabolites in the samples. Concurrently, the mass spectrometry data were compared with publicly available metabolic databases such as HMDB (http://www.hmdb.ca/, accessed on 5 April 2024) and Metlin (https://metlin.scripps.edu/, accessed on 5 April 2024), as well as MeiJi’s custom libraries, to obtain comprehensive metabolite information. Ion peaks exhibiting more than 50% missing values within the group were excluded from subsequent statistical evaluations to ensure the integrity of the analysis. The data were normalized by total peak area and subjected to Log_10_ transformation. The ion peaks were integrated, and Python software version 3.12 was utilized for pattern recognition. Additionally, the data were pre-processed using unit variance scaling (UV) to facilitate subsequent data analysis. PCA provided insights into the differences in overall metabolites and the variations among samples. HCA was employed to investigate patterns of metabolite accumulation under various conditions, which were visualized through heatmaps accompanied by dendrograms. Subsequently, metabolites were evaluated using FC values, adhering to the screening criteria of |FC| ≥ 2, *p* ≤ 0.05, and VIP ≥ 1.

### 2.5. Preparation and Calculation of Amino Acid Metabolites Content Standard Curve

The process involved preparing standard solutions with varying concentrations (10, 20, 50, 100, 200, 500, 1000, 2000, 5000, 10,000, and 20,000 ng/mL) and collecting mass spectral peak intensity data for each concentration. The concentration ratio between the external standard and the internal standard was plotted on the x-axis, while the peak area ratio (area ratio) between the external and internal standards was represented on the y-axis. This approach facilitated the creation of standard curves for different compounds. Quantitative formulas for all 94 amino acid metabolites were established (Table S3). The process of determining the levels of amino acid metabolites required substituting the ratio of the integrated peak area from the identified samples into the standard curve’s linear equation for calculation. This equation was subsequently utilized in a calculation method to derive the substance content data (*Y*) in the actual samples.

## 3. Results

### 3.1. Characterization of Amino Acid Metabolic Profiles

In this study, the LC-MS/MS method was employed for targeted metabolomics analysis, facilitating the quantification of 94 amino acids (Table S4). An examination of the total ion chromatogram (TIC) from the QC and 12 samples revealed significant curve alignment, suggesting the presence of various metabolites (Figures S1 and S2) and confirming the reliable performance of the instrument in metabolite detection.

### 3.2. Principal Component Analysis (PCA) and Hierarchical Cluster Analysis (HCA)

**Figure 1:**
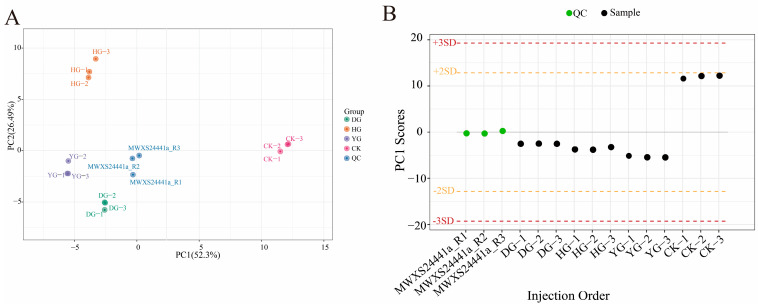
(**A**) PCA scores for each group. The horizontal axis represents PC1, while the vertical axis represents PC2, which denotes the two directions exhibiting the greatest variation in the data. The accompanying percentage indicates the proportion of variation in each direction relative to the total variation. The dots in the graph correspond to different samples, with the colors and shapes signifying distinct groups. The circles of varying colors illustrate the clustering patterns among the different groups, and the size of the circles typically reflects the dispersion of samples within each group; (**B**) Overall sample PC1 control chart (for testing data accuracy). The horizontal coordinate represents the sample name, while the vertical coordinate indicates the value of PC1.

A total of 15 samples, including 3 QC samples and 12 *O. sinensis* samples, were analyzed using PCA to gain a preliminary understanding of the overall metabolic differences between groups and the degree of variability among samples within each group (Figure 1A). The PCA plot effectively illustrates the metabolic differences and variations among samples within each group. The top two principal components accounted for 52.3% and 26.49% of the variance. The PCA analysis revealed that the DG, YG, and QC clusters were closely related, while the HG cluster was distinctly separated, indicating notable variations in metabolite composition. The reliability of the testing procedure was confirmed by evaluating QC samples using a standardized PCA model. The PC1 values remained within the range of plus or minus 3 standard deviations (SD), consistent with the expected values (Figure 1B), thereby validating the quality of the data.

**Figure 2:**
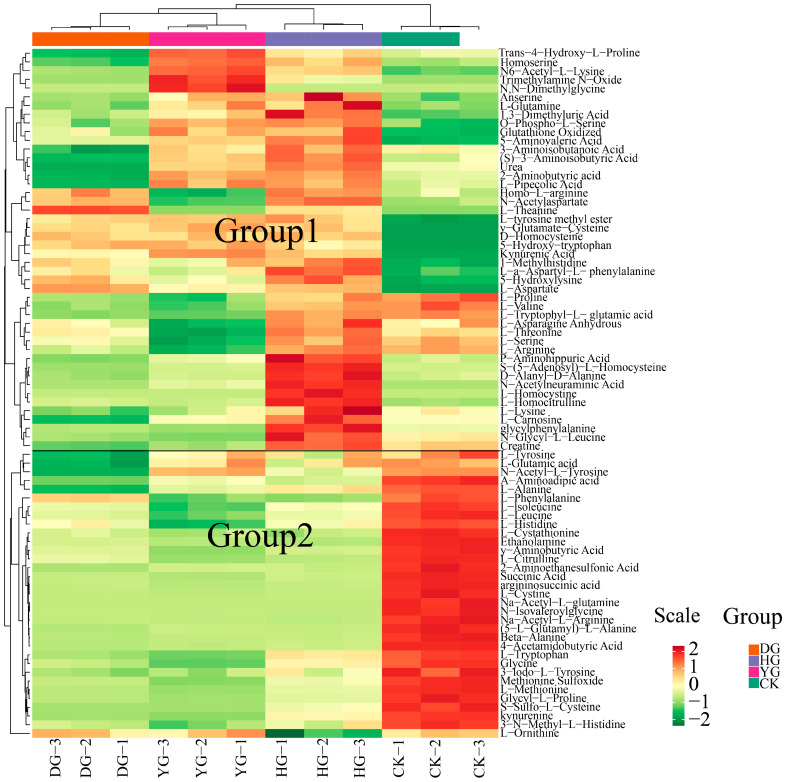
HCA of amino acid content between *O. sinensis* samples is presented. The left side of the figure displays the names of the samples, while the right side presents a dendrogram illustrating the clustering of these samples. The upper section features the dendrogram for metabolite clustering, and the lower section lists the names of the metabolites. The proximity of the sample branches indicates the similarity in the cumulative patterns of all metabolites between the two samples. In the figure, red indicates metabolites with higher expression levels in the sample, whereas green denotes those with lower expression levels.

To illustrate the categorization of 79 amino acids across various drying scenarios, a heat map of amino acid metabolites is presented in Figure 2. The findings from HCA revealed that the metabolites clustered into two distinct groups: one group consisting of 46 metabolites, and the other included 33 metabolites. Notably, the metabolites of *O. sinensis* subjected to different drying methods exhibited contrasting accumulation patterns compared to those of fresh *O. sinensis*. The heatmap generated through hierarchical cluster analysis delineated the interrelations among the sample clusters, corroborating the findings of the PCA. In summary, the amino acid metabolites of the DG, YG, and HG cohorts coalesced, indicating further distinction within the DG and YG subclasses, while the CK group formed a separate distinct cluster.

### 3.3. Calculation of Amino Acid Metabolites

The analysis of the data was conducted using the MultiQuant 3.0.3 application. Calibration of the chromatographic peaks was performed by utilizing information on peak patterns and retention times (RT) obtained from the standard, aiming to achieve accurate qualitative and quantitative results. The peak area data and standard curve equation were referenced (Table 1) to quantify the levels of amino acid metabolites in the samples.

An examination of 94 metabolites derived from amino acids revealed that fifteen specific amino acids, including phosphorylethanolamine, nicotinuric acid, and N8-acetylspermidine, could not be detected. Among the 94 detected amino acid metabolites, the ten with the highest absolute concentrations were L-glutamic acid, oxidized glutathione, L-ornithine, L-aspartate, argininosuccinic acid, L-threonine, L-arginine, and L-histidine. *O. sinensis* exhibits varying amounts of amino acid metabolites depending on the drying method used, with L-glutamic acid being the most abundant, particularly in the YG group. The hierarchical cluster analysis heatmap (see Figure 2 for details) demonstrated that the relationships among the sample clusters were consistent with the PCA results (see Figure 1A for details). In summary, the amino acid metabolites of the DG, YG, and HG groups clustered together, with further analysis revealing subclasses within the DG and YG groups, while the CK group formed a distinct cluster.

#### Difference Analysis of Essential Amino Acid Metabolites and Delicious Amino Acids Metabolites Content

**Figure 3:**
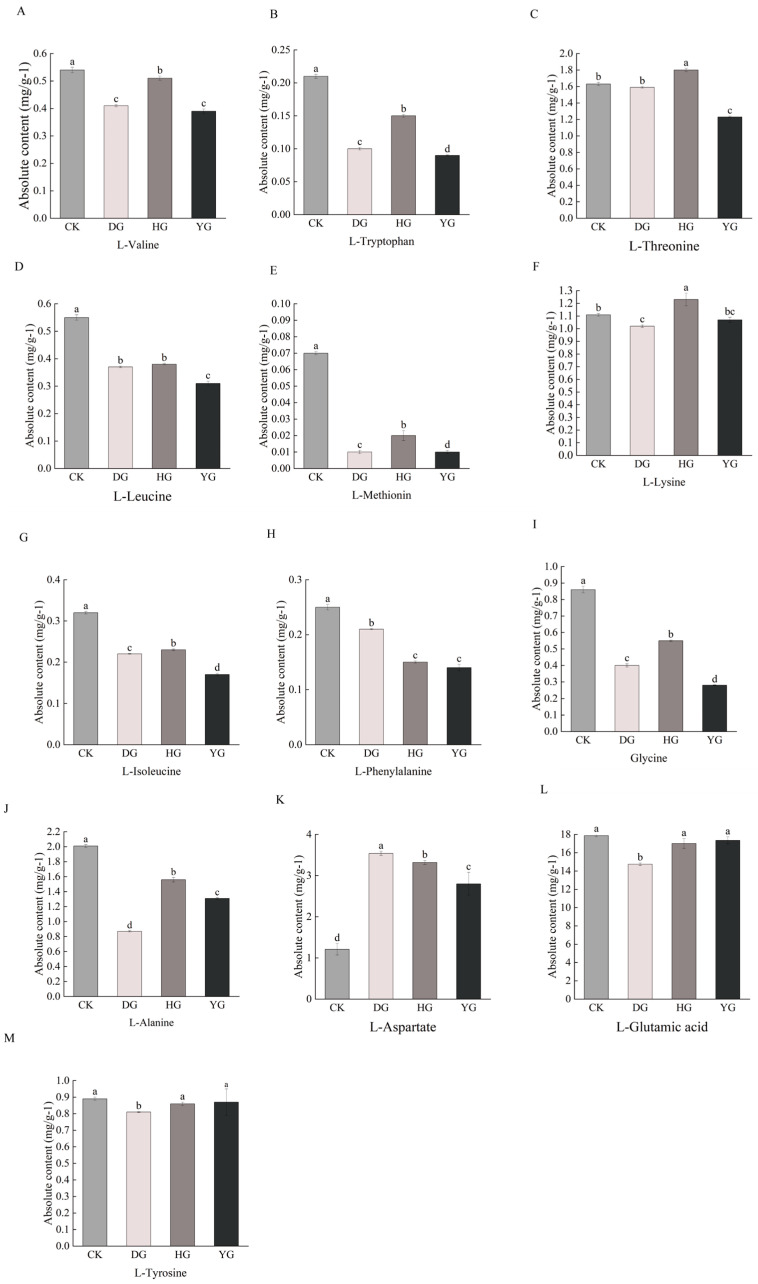
(**A**–**M**) Difference analysis of essential amino acid and delicious amino acids metabolites content. This study employs a one-way ANOVA to analyze essential and tasty amino acids. The horizontal axis denotes group names, while the vertical axis illustrates the metabolite contents of amino acids. Distinct lowercase letters indicate significant differences in amino acid contents among the samples (*p* < 0.05).

Amino acids play a crucial role in sustaining human existence. Proteogenic amino acids are essential for both humans and animals, as they cannot be synthesized independently by the body [29]. Amino acids serve various physiological roles and can exhibit sweet, bitter, fresh, or sour flavors [30]. Research has identified a total of 79 amino acid metabolites, which include eight essential amino acids: L-phenylalanine, L-threonine, L-tryptophan, L-lysine, L-methionine, L-isoleucine, L-leucine, and L-valine. Additionally, six amino acids known for their flavorful properties include glutamic acid, aspartic acid, phenylalanine, alanine, glycine, and tyrosine. Comparative analysis revealed statistically significant differences (*p* < 0.05) in the concentrations of essential and flavorful amino acids in *O. sinensis*, depending on the drying methods employed (Figure 3A–M). Among the eight essential amino acids except L-threonine and L-Lysine, the contents of the other six amino acids in the CK group were higher than those of *O. sinensis* under the other three drying methods. Among the delicious amino acids, except for L-aspartate, the contents of the other five amino acids in the CK group were higher than those of *O. sinensis* under the other three drying methods. The effects of various drying methods on the content of essential amino acid metabolites and delicious amino acid metabolites were inconsistent. However, fresh *O. sinensis* (CK) exhibited a higher content of both essential and delicious amino acid metabolites compared to the other three groups, with the lowest levels observed in the HG group.

### 3.4. Screening of Differential Amino Acid Metabolites (DAMs)

**Figure 4:**
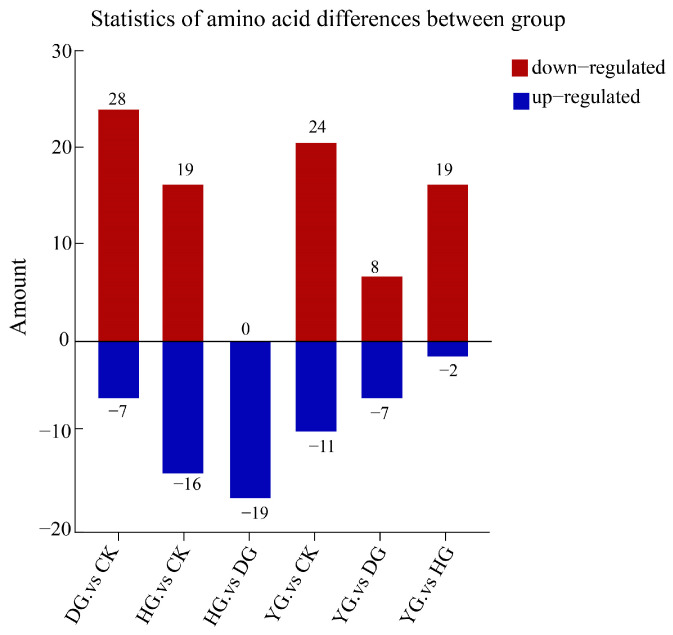
Statistics of amino acid metabolites differences between groups. The horizontal axis represents between-group comparisons, while the vertical axis indicates the number of metabolites. Red denotes up-regulated expression, whereas blue indicates down-regulated expression.

**Figure 5:**
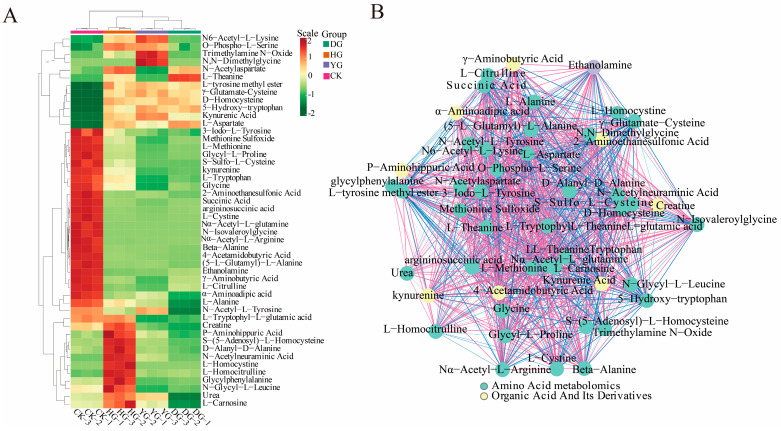
(**A**) Heat map depicting 48 significantly poor metabolite clusters. The horizontal axis represents the sample names, while the upper section illustrates the clustering of these samples. The right side lists the names of the metabolites, and the left side displays the clustering of the metabolites. (**B**) Schematic representation of the correlation network for significantly different metabolites. The dots in the graph represent metabolites that exhibit significant differences. The color of each dot corresponds to the category of the metabolite; the red lines indicate positive correlations, while the blue lines signify negative correlations.

In this study, we analyzed 79 amino acid components in *O. sinensis* using various drying methods, with a focus on group-specific quantitative metabolite multipliers. The differential multipliers of the metabolites were calculated using a base 2 logarithm (Log_2_FC). We specifically examined metabolites that met the criteria for differential amino acids, defined as having |FC| ≥ 2, *p* ≤ 0.5, and VIP ≥ 1. The results revealed 48 metabolites with significant differences, including (5-l-glutamyl)-l-alanine, 2-aminoethanesulfonic acid, 3-iodo-l-tyrosine and 4-acetamidobutyric acid, etc. (see Table S5). A comparative analysis of the amino acid metabolites of *O. sinensis* under different drying methods indicated variations in the number of metabolites between groups (Figure 4). The heat map presented in Figure 5A illustrates the clustering of various amino acid metabolites, revealing distinct characteristics among the samples. The samples were approximately grouped into four clusters, with DG, YG, and HG forming sub-branches. The CK and HG groups were more pronounced in the between-group comparison. The CK group clustered together, characterized by higher levels of 2-aminoethanesulfonic acid, L-cystine, and 3-iodo-l-tyrosine, and lower levels of D-homocysteine. HG samples had relatively high levels of glycylphenylalanine, D-alanyl-d-alanine, and L-homocystine, while ethanolamine and L-citrulline levels were lower.

**Figure 6:**
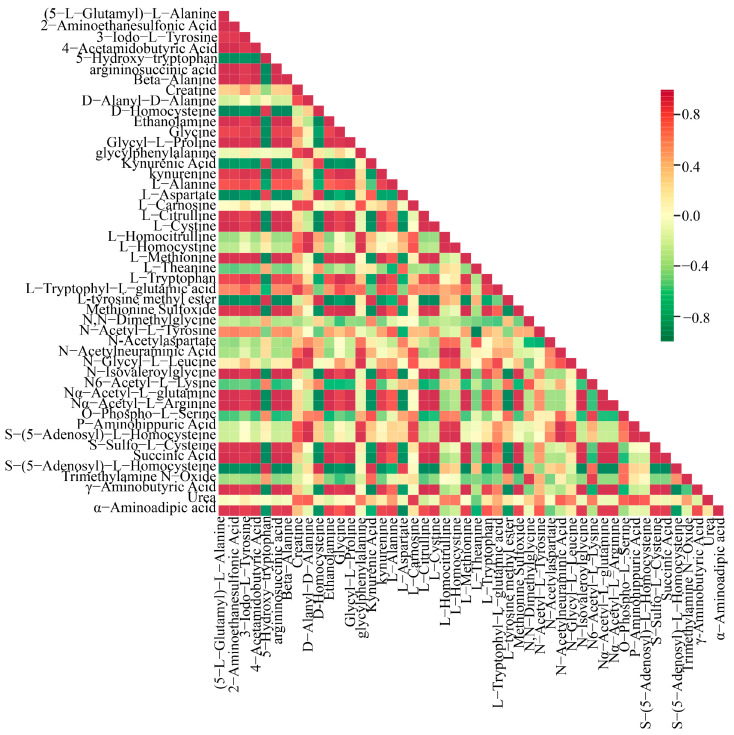
Pearson correlation analysis among DG, HG, YG, and CK is shown in the correlation plot. The red squares in the graph indicate greater correlation and the green squares indicate lesser correlation.

A Pearson correlation analysis was conducted on a selected group of 48 amino acids exhibiting significant variations. The findings were visually represented through a correlation network diagram (Figure 5B) as well as a correlation heat map (Figure 6). Additionally, a violin chart was employed to display the distribution of data and likelihood density, merging elements of a boxplot and density map. In this graphical representation, the range of quartiles is depicted by the central box, while the 95 % confidence interval is denoted by the slender black line that extends from it. The median is represented by the horizontal line at the center, and the density distribution of the data is illustrated by the outer shape (Figure S3). These findings illuminate the connections among diverse amino acids and provide insights into the reaction mechanisms of *O. sinensis* under various drying techniques. The reliability and robustness of these data have been confirmed. Furthermore, the results highlighted significant variations in the accumulation and reduced expression of crucial amino acids, including phenylalanine, tryptophan, leucine, isoleucine, and valine, among the different experimental groups.

### 3.5. KEGG Analysis

**Figure 7:**
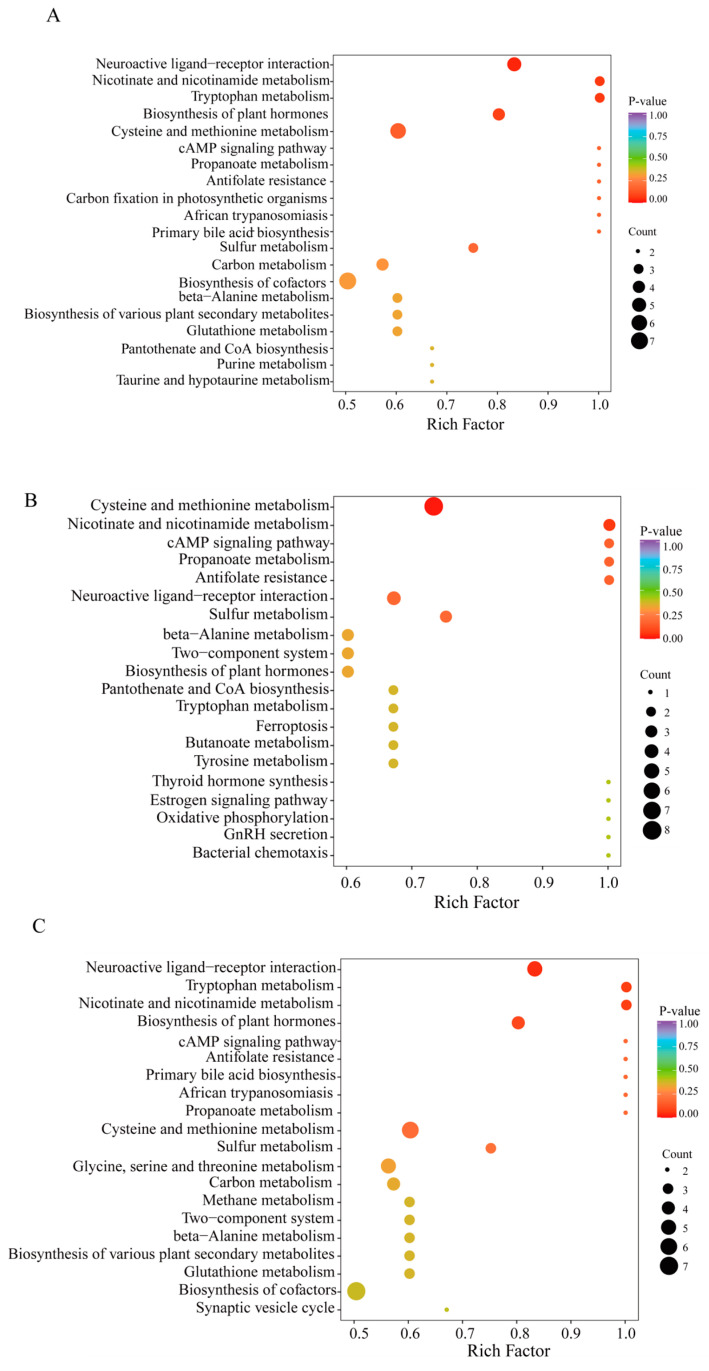
Enrichment of metabolites using KEGG pathways (**A**–**C**). (**A**): DG vs. CK. (**B**): HG vs. CK, (**C**): YG vs. CK. The horizontal axis represents the enrichment factor for each metabolic pathway, whereas the vertical axis lists the names of the metabolic pathways. The color of the circles reflects the *p*-value, with redder hues indicating a greater degree of enrichment. Furthermore, the size of each circle is proportional to the number of enriched metabolites.

The KEGG annotation findings for notably distinct metabolites were organized to investigate the roles and pathways of different metabolites in *O. sinensis* subjected to various drying techniques based on their classifications within KEGG pathways (Figures S4–S6). The number of distinct metabolites in pathways, such as the biosynthesis of secondary metabolites, amino acids, cysteine, methionine, and other secondary metabolites, as well as cofactor biosynthesis pathways, was notably high across all three comparison groups (Figure 7A–C). Within genetic information processing, 5, 3, and 5 amino acid metabolites were linked to aminoacyl-tRNA biosynthesis in the DG. vs. CK, HG. vs. CK, and YG. vs. CK groups, respectively. In the EIP classification, amino acid metabolites, specifically 6, 3, and 4, were linked to ABC transporters in the DG compared to CK, HG compared to CK, and YG compared to CK groups, respectively. Additionally, an analysis of amino acid metabolites enrichment in the KEGG database revealed noteworthy pathways, including neuroactive ligand-receptor interaction, metabolism of cysteine and methionine, and synthesis of plant hormones across the three comparison samples. Interestingly, the biosynthesis of cofactors was a notable enrichment pathway in the DG. vs. CK and YG. vs. CK groups but was absent in the HG. vs. CK group.

### 3.6. DAMs Analysis in Critical Paths

**Figure 8:**
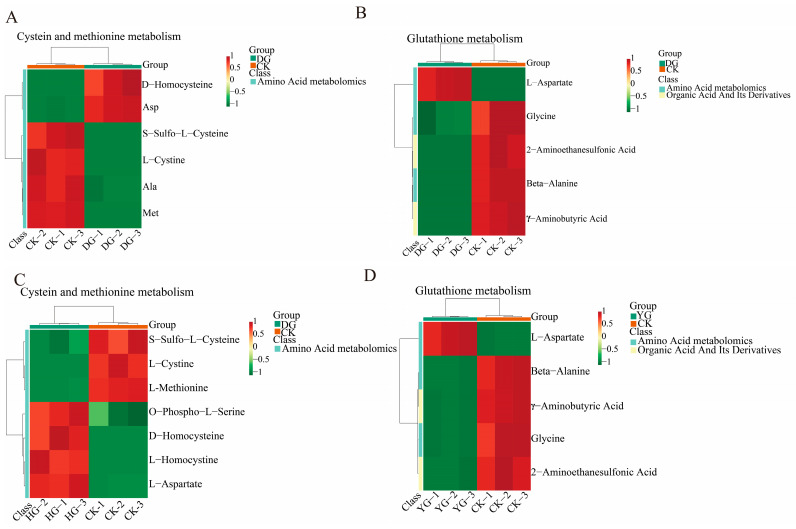
(**A**–**D**) This heatmap illustrates the clustering of differential metabolites associated with the KEGG pathway. The names of the metabolites are displayed on the left, while the sample names are shown along the bottom. Red indicates up-regulation, green indicates down-regulation.

Using the KEGG annotation information of metabolites identified according to the screening criteria (Figure 8A–D), we selected two key differential metabolic pathways for further analysis. All metabolites within these pathways were clustered to enhance our understanding of the changing patterns of substance contents in potentially important metabolic pathways across different subpopulations. Comparisons among the various groups reveal divergent trends in metabolite levels, indicating that the metabolite compositions of *O. sinensis* were significantly influenced by the different drying methods employed. Metabolites involved in the metabolism of cysteine and methionine, as well as those from the glutathione metabolic pathway, serve as crucial indicators for distinguishing the quality of *O. sinensis* across various drying techniques. Notable examples include L-aspartate, L-cysteine, glycine, and L-methionine.

## 4. Discussion

Metabolomics is a systems biology approach that emphasizes the development and evaluation of comprehensive biochemical analysis techniques for metabolites in biological systems [31]. This study utilized LC-MS/MS metabolomics to measure and analyze amino acids and their metabolites in *O. sinensis*, employing various drying methods. The results indicated that the effects of different drying methods on the content of amino acid metabolites varied significantly, with L-glutamic acid exhibiting the highest concentration. L-glutamic acid has been shown to provide benefits for human metabolism in various studies without any associated risk of bodily accumulation [32,33].

L-glutamic acid is primarily utilized in the production of monosodium glutamate and various spices. Additionally, it serves as a salt substitute, nutritional supplement, and biochemical reagent, among other applications [34,35]. As a compound, L-glutamic acid functions as a medication, playing a role in the metabolism of proteins and sugars within the brain, thereby enhancing oxidative processes. This process results in the formation of non-toxic glutamine from ammonia in the body, which helps to reduce blood ammonia levels and alleviate symptoms associated with hepatic coma [36]. It is chiefly employed in the management of hepatic coma and severe hepatic insufficiency, among other conditions [37]. The levels of L-glutamic acid in the YG, HG, and CK groups exhibited no significant variation among these groups (*p* > 0.05) but were notably higher than those in the DG group (see Table 1 for details). L-glutamine, one of the amino acids, is predominantly synthesized in muscle and is essential for various biological functions, including protein synthesis, regulation of renal and immune system activities, and maintenance and repair of intestinal tissues [38,39,40]. Moreover, it serves as a crucial precursor for compounds in the agricultural, chemical, and pharmaceutical sectors, presenting significant market potential [41,42,43]. There were no significant differences in the levels of L-glutamine between the YG and HG groups (*p* > 0.05) (see Figure 3L for details). However, the levels in the YG group were significantly higher compared to those in the DG and CK groups. A KEGG enrichment analysis indicated that the various metabolites within the groups were predominantly associated with pathways such as methionine and cysteine metabolism, the synthesis of plant-related hormones, and the interactions between neuroactive receptors and their ligands (see Figure 7 for details). This analysis suggested that these three metabolic pathways influenced the amino acid metabolite content and protein synthesis of *O. sinensis* under different drying methods.

Metabolites, which are formed following gene expression, transcription, and protein synthesis, are typically influenced by both environmental and genetic factors [44,45]. When subjected to heat and dehydration, amino acids and sugars can degrade thermally, undergo Maillard reactions, and experience lipid oxidation, resulting in the formation of volatile compounds such as alcohols, aldehydes, and ester flavors [46,47]. The variations observed in amino acids and their metabolites in *O. sinensis* across the three drying methods—DG, HG, and YG—may stem from the complex interplay of these enzymatic, oxidative, and thermal reactions.

Observations revealed complex changes in the content of amino acid metabolites across samples subjected to various drying methods (see Figure 6 for details). These variations exhibited distinct patterns, influenced by both the type of amino acid metabolites and the drying technique employed. Cluster analysis of 48 significantly different metabolites indicated that L-tryptophyl-L-glutamic acid, creatine, P-aminohippuric acid, S-(S-adenosyl)-L-homocysteine, D-alanyl-d-alanine, N-acetylneuraminic acid, L-homocystine, L-homocitrulline, glycylphenylalanine, N-glycy-L-leucine, urea, and L-carnosine were notably increased by the HG method, suggesting a response to the high-temperature drying process. Among the three drying methods yielding the highest amino acid metabolite contents, L-glutamic acid and oxidized glutathione exhibited a similar increasing trend. In contrast, the L-glutamic acid content in the samples showed complex fluctuations during the drying process. The DG approach may damage tissue cell ultrastructure due to ice crystal formation, leading to the release of enzymes and pro-oxidants that contribute to protein denaturation and degradation. This mechanism might account for the observed decrease in amino acid metabolite levels during the freezing stages [48].

Essential amino acids are those that the human body cannot produce independently, or if they are produced, they are synthesized at a rate insufficient to meet the organism’s requirements [49]. These amino acids must be obtained from dietary proteins to maintain normal metabolism, homeostasis, and overall health. The levels of crucial amino acids in *O. sinensis* varied significantly depending on the drying method employed, with elevated levels observed under the DG and HG methods compared to the YG method. Related studies have explored the effects of various drying techniques on the chemical composition and amino acid profile of sea cucumbers. The results indicated that freeze drying (MD) is more effective in preserving specific essential amino acids, a finding that corroborates the results of the present study [50]. However, when considering a particular class of essential amino acids, it is important to evaluate different drying methods.

The content of amino acid metabolites plays a crucial role in regulating both the flavor and unique scent of the organism [51]. The ‘fishy’ smell associated with other fungi of *O. sinensis* arises from various factors, primarily different lipids, ketones, and aldehydes. However, *O. sinensis* is particularly rich in glutamic acid, aspartic acid, phenylalanine, and other amino acids that contribute to its distinctive flavor. In this study, we plotted histograms illustrating the content of flavor-contributing amino acids in *O. sinensis* under various drying methods. The results indicated a significant decrease in the content of flavor-contributing amino acids across the different drying methods (see Figure 3 for details). Related studies have examined the impact of drying methods on the aroma and fresh flavor of Dendrobium officinale flower tea, revealing that amino acid metabolites are closely associated with the fresh flavor of this tea, which aligns with the findings of the current study [52]. Future research should delve deeper into the mechanisms by which different drying methods affect the fresh flavor of *O. sinensis.*

The present study offers a novel and comprehensive metabolic analysis of 94 amino acids found in *O. sinensis* subjected to various drying methods. The analysis reveals elevated levels of L-glutamic acid and L-glutamic in *O. sinensis*. This significant data enhances the understanding of the amino acids’ metabolite in *O. sinensis* following different drying techniques. However, this study only describes the effects of various drying methods on *O. sinensis* at the level of amino acid metabolic profile characteristics. The results are somewhat limited and should be analyzed in conjunction with other histological, physiological, and biochemical indices in future research.

## 5. Conclusions

Using LC-MS/MS, this research successfully quantified a total of 94 amino acid metabolites. Of these, 79 metabolites were identified in *O. sinensis*, while 15 metabolites were not detected. Among the identified metabolites, L-glutamic acid and L-glutamine exhibited particularly high levels, followed by oxidized glutathione, L-ornithine, aspartate, and γ-aminobutyric acid, among others. Notably, glutamic acid (Glu) and aspartic acid (Asp) are hydrophilic amino acids, which are crucial for their freshness properties. Among the 79 amino acids and their metabolites identified, 48 showed significant differences. Principal component analysis indicated a strong similarity between the DG and YG groups, while the HG group displayed considerable separation from the others, suggesting distinct metabolite compositions. Analysis of essential and delicious amino acid metabolites revealed that fresh *O. sinensis* (CK) exhibited a higher content of both essential and palatable amino acid metabolites than the other three groups. Cluster analysis revealed a reduction in the accumulation of primary amino acid metabolite compounds in both the YG and HG categories, likely due to changes in the C/N ratio of *O. sinensis* resulting from temperature fluctuations, which affected nutrient uptake and utilization. The findings of this study suggest that the drying method significantly influences the amino acid metabolite content of *O. sinensis*, with a more pronounced effect noted on essential and flavor-related amino acids, thereby affecting its overall quality. However, a comprehensive evaluation of *O. sinensis* under various drying methods should also consider other active ingredients and genome sequencing to thoroughly examine its metabolic potential and address quality concerns.

## Figures and Tables

**Table 1:** Quantitative detection results of 94 amino acids metabolites (ng/g).

No.	Compounds	Q1 (Da)	Q3 (Da)	Molecular Weight	Formula	RT	DG	HG	YG	CK
1	Ethanolamine	62	44	61.08	C_2_H_7_NO	4.73	52,263.13	30,905.25	27,724.37	34,209.60
2	Phosphorylethanolamine	142.026	44.049	141.06	C_2_H_8_NO_4_P	N/A	N/A	N/A	N/A	N/A
3	Anserine	241	170	240.26	C_10_H_16_N_4_O_3_	10.47	2791.09	3269.22	2023.59	3102.44
4	(5-L-Glutamyl)-L-Alanine	219.09	202	218.09	C_8_H_14_N_2_O_5_	9.84	59,690.84	152,090.55	70,597.08	174,067.24
5	O-Phospho-L-Serine	186	88.2	185.01	C_3_H_8_NO_6_P	10.92	5014.20	10,415.13	5146.75	9795.23
6	N-Acetylaspartate	176	134	175.05	C6H9NO5	4.76	18,063.90	20,873.08	12,980.58	8152.68
7	N-Acetyl-L-Tyrosine	224	136	223.09	C_11_H_13_NO_4_	1.74	680.43	1210.69	630.95	1665.39
8	L-tyrosine methyl ester	196.2	136.3	195.09	C_10_H_13_NO_3_	1.52	409.23	434.79	281.85	437.05
9	L-Cystathionine	223.07	134	222.07	C_7_H_14_N_2_O_4_S	10.86	409,853.38	386,011.47	265,291.90	383,347.89
10	Homoserine	120.065	74.06	119.12	C_4_H_9_NO_3_	8.57	963,95.82	160,750.02	85,718.14	181,130.92
11	D-Alanyl-D-Alanine	161.09	44.04	160.09	C_6_H_12_N_2_O_3_	8.76	3157.84	7888.17	3684.92	3824.47
12	S-(5-Adenosyl)-L-Homocysteine	385	136.06	384.12	C_14_H_20_N_6_O_5_S	9.37	115,970.38	835,272.49	317,084.08	224,819.03
13	3-Iodo-L-Tyrosine	308.2	261.97	306.97	C_9_H_10_INO_3_	4.75	37.26	66.00	36.00	42.47
14	glycylphenylalanine	223.2	120.2	222.1	C_11_H_14_N_2_O_3_	7.17	881.62	7363.57	2750.79	417.58
15	L-Valine	118.1	72.1	117.08	C_5_H_11_NO_2_	6.14	405,663.10	512,162.35	305,943.86	389,962.93
16	L-Tyrosine	182.08	136.07	181.19	C_9_H_11_NO_3_	6.67	808,353.61	869,834.54	559,398.27	872,333.69
17	L-Tryptophan	205.1	118.06	204.09	C_11_H_12_N_2_O_2_	4.46	101,179.10	151,314.67	84,166.08	86,786.14
18	Trans-4-Hydroxy-L-Proline	132.058	86	131.06	C_5_H_9_NO_3_	8.05	98,744.37	156,291.27	85,014.56	191,836.09
19	Glutathione Oxidized	613.152	484	612.15	C_20_H_32_N_6_O_12_S_2_	11.31	7,287,906.55	8,895,298.80	5,394,405.55	8,784,995.30
20	argininosuccinic acid	291.5	176.1	290.27	C_10_H_18_N_4_O_6_	10.99	2,964,334.61	3,027,278.70	1,997,208.10	1,930,567.70
21	D-Homocysteine	136	90.036	135.04	C_4_H_9_NO_2_S	6.14	464.30	486.99	319.14	452.44
22	L-Cystine	239	120	240.02	C_6_H_12_N_2_O_4_S_2_	10.91	401.06	594.09	335.35	N/A
23	Nicotinuric Acid	181.1	135	180.05	C_8_H_8_N_2_O_3_	3.55	N/A	N/A	N/A	N/A
24	N8-Acetylspermidine	190.18	173.15	189.18	C_9_H_23_N_3_O	9.72	N/A	N/A	N/A	N/A
25	N-Propionylglycine	130.1	74	131.06	C_5_H_9_NO_3_	2.14	N/A	N/A	N/A	N/A
26	L-Cysteine	122.02	59	121.02	C_3_H_7_NO_2_S	7.13	N/A	N/A	N/A	N/A
27	3-Chloro-L-Tyrosine	216.1	170.1	215.04	C_9_H_10_C_l_NO_3_	5.33	N/A	N/A	N/A	N/A
28	5-Hydroxy-Tryptamine	177.1	160	176.21	C_10_H_12_N_20_	2.55	N/A	N/A	N/A	N/A
29	Nα-Acetyl-L-glutamine	189.1	130.1	188.18	C_7_H_12_N_2_O_4_	6.39	N/A	N/A	N/A	N/A
30	L-Threonine	120.06	74	119.06	C_4_H_9_NO_3_	8.38	1,590,252.44	1,797,878.87	1,129,379.90	1,226,147.51
31	N,N-Dimethylglycine	104	58	103.06	C_4_H_9_NO_2_	5.91	N/A	N/A	N/A	566.57
32	L-Homocystine	269	136	268.06	C_8_H_16_N_2_O_4_S_2_	10.50	N/A	3648.26	N/A	N/A
33	Trimethylamine N-Oxide	76	58.1	75.07	C_3_H_9_NO	2.94	N/A	3.87	N/A	13.15
34	N-Isovaleroylglycine	158.1	74	159.09	C_7_H_13_NO_3_	1.32	N/A	N/A	N/A	N/A
35	S-Sulfo-L-Cysteine	201.98	120	200.98	C_3_H_7_NO_5_S_2_	7.86	N/A	1428.78	N/A	N/A
36	N-Acetylneuraminic Acid	310.11	274.09	309.11	C_11_H_19_NO_9_	9.57	N/A	130,727.88	N/A	22,726.06
37	γ-Glutamate-Cysteine	251.07	130.05	250.06	C_8_H_14_N_2_O_5_S	9.84	207,439.45	215,940.92	141,130.07	244,866.08
38	5-Hydroxy-tryptophan	219.1	74	220.22	C_11_H_12_N_2_O_3_	6.69	15,855.02	13,153.19	9671.64	16,623.56
39	L-Theanine	175	158	174.1	C_7_H_14_N_2_O_3_	7.14	2049.75	1054.52	1037.14	N/A
40	L-Tryptophyl-L-glutamic acid	334	159	333.13	C_16_H_19_N_3_O_5_	8.19	52,085.37	235,428.36	95,840.64	34,439.12
41	Urea	61	44	60.03	CH_4_N_2_O	1.46	73,727.18	380,087.66	151,272.10	304,614.48
42	L-Serine	106.04	60	105.04	C_3_H_7_NO_3_	9.03	590,199.33	684,869.12	425,025.83	474,754.60
43	L-Histidine	156.07	110.07	155.07	C_6_H_9_N_3_O_2_	10.06	1,405,379.02	1,404,476.98	936,622.02	1,071,662.56
44	Glycyl-L-Proline	173.1	116.071	172.09	C_7_H_12_N_2_O_3_	9.10	788.76	1743.52	847.13	285.07
45	Glycine	76.03	30	75.03	C_2_H_5_NO_2_	8.6	403,027.10	553,800.33	318,945.34	282,386.73
46	Succinic Acid	117.03	99	118.03	C_4_H_6_O_4_	1.36	1,299,153.87	1,491,250.31	930,135.18	730,942.74
47	L-Glutamine	147.069	84	146.07	C_5_H_10_N_2_O_3_	8.98	9,799,945.11	11,662,924.50	7,154,292.86	11,198,431.33
48	Beta-Alanine	90.2	30	89.05	C_3_H_7_NO_2_	8.05	43,449.96	60,525.95	34,661.32	54,251.24
49	L-α-Aspartyl-L-phenylalanine	281.3	166	280.11	C_13_H_16_N_2_O_5_	8.73	3981.51	5021.82	3004.02	3711.77
50	L-Aspartate	134.04	74	133.1	C_4_H_7_NO_4_	9.93	3,544,943.12	3,319,674.22	2,288,209.09	2,792,831.29
51	L-Asparagine Anhydrous	133.06	74	132.05	C_4_H_8_N_2_O_3_	9.13	895,965.13	974,125.64	623,366.63	815,367.47
52	L-Arginine	175.1	70.06	174.11	C_6_H_14_N_4_O_2_	10.28	1,231,313.96	1,537,085.89	922,803.38	1,047,209.36
53	L-Alanine	90.05	44.05	89.05	C_3_H_7_NO_2_	8.07	866,190.62	1,556,109.93	807,436.21	1,313,825.59
54	5-Hydroxylysine	163.1	128.07	162.18	C_6_H_14_N_2_O_3_	10.76	13,266.38	14,017.87	9098.34	12,117.00
55	3-N-Methyl-L-Histidine	170	96	169.18	C_7_H_11_N_3_O_2_	10.19	31,543.58	32,713.75	21,422.51	30,344.09
56	3-Aminoisobutanoic Acid	104.07	86.05	103.06	C_4_H_9_NO_2_	6.92	165,603.26	257,073.67	140,894.62	232,608.79
57	1-Methylhistidine	170.1	124	169.09	C_7_H_11_N_3_O_2_	9.40	224,915.30	237,642.61	154,189.10	220,762.47
58	Homo-L-arginine	189	144	188.13	C_7_H_16_N_4_O_2_	10.14	399,822.88	407,488.07	269,107.03	329,249.02
59	L-Isoleucine	132.1	86.1	131.1	C_6_H_13_NO_2_	4.67	220,177.26	229,017.66	149,733.20	167,765.33
60	L-Glutamic acid	148.06	84	147.05	C_5_H_9_NO_4_	9.39	14,736,617.03	17,017,140.07	10,584,588.83	17,347,695.43
61	L-Citrulline	176.1	113	175.1	C_6_H_13_N_3_O_3_	9.37	210,279.70	144,586.95	118,292.00	103,110.97
62	L-Proline	116.1	70.1	115.06	C_5_H_9_NO_2_	6.20	1,026,738.00	1,117,680.18	714,808.13	1,002,877.35
63	L-Phenylalanine	166.1	120.08	165.08	C_9_H_11_NO_2_	4.29	212,228.29	147,826.34	120,019.64	139,718.17
64	Nα-Acetyl-L-Arginine	217.1	158	216.12	C_8_H_16_N_4_O_3_	8.40	4080.90	3937.66	2675.65	4013.49
65	L-Carnosine	227.2	156	226.11	C_9_H_14_N_4_O_3_	10.00	7058.33	16,999.45	8022.59	11,899.32
66	N-Glycyl-L-Leucine	189.1	86	188.12	C_8_H_16_N_2_O_3_	7.12	3348.98	23,136.08	8830.73	1577.20
67	Methionine Sulfoxide	166	74	165.05	C_5_H_11_NO_3_S	8.95	2511.90	3535.30	2018.72	1485.36
68	L-Methionine	150.05	104	149.05	C_5_H_11_NO_2_S	5.49	12,821.99	21,771.01	11,532.83	7682.36
69	L-Lysine	147.069	84	146.11	C_6_H_14_N_2_O_2_	10.45	1,016,825.72	1,230,450.69	749,095.62	1,073,002.26
70	N6-Acetyl-L-Lysine	189.1	126	188.12	C_8_H_16_N_2_O_3_	8.32	142,014.00	207,737.40	116,586.57	252,095.63
71	Sarcosine	90.2	44.1	89.05	C_3_H_7_NO_2_	7.51	N/A	N/A	N/A	N/A
72	L-Leucine	132.1	86.1	131.1	C_6_H_13_NO_2_	4.37	371,341.70	383,117.21	251,487.76	307,224.27
73	L-Pipecolic Acid	130	84	129.08	C_6_H_11_NO_2_	6.58	10,013.19	13,560.04	7859.94	13,645.17
74	L-Ornithine	133.09	70	132.16	C_5_H_13_C_l_N_2_O_2_	10.50	6,438,468.41	5,370,492.12	3,936,323.68	6,452,913.39
75	L-Homocitrulline	190.1	127	189.11	C_7_H_15_N_3_O_3_	9.14	8630.02	20,657.64	9765.60	8303.07
76	(S)-β-Aminoisobutyric Acid	104.07	57.03	103.06	C_4_H_9_NO_2_	6.92	142,049.24	232,551.66	124,869.27	207,825.77
77	1,3-Dimethyluric Acid	195	180	196.06	C_7_H_8_N_4_O_3_	1.53	945.92	1515.58	821.01	1196.77
78	Kynurenic Acid	190.043	144	189.04	C_10_H_7_NO_3_	3.02	70.73	86.35	53.37	102.69
79	N’-Formylkynurenine	237.1	146.06	236.08	C_11_H_12_N_2_O_4_	5.47	N/A	N/A	N/A	N/A
80	Guanidinoethyl Sulfonate	168.04	151.01	167.04	C_3_H_9_N_3_O_3_S	6.37	N/A	N/A	N/A	N/A
81	2-Aminobutyric acid	104.1	58.14	103.12	C_4_H_9_NO_2_	7.25	217,013.31	413,898.45	210,306.34	403,754.53
82	Creatine Phosphate	212.04	114.06	211.11	C_4_H_8_N_3_Na_2_O_5_P	N/A	N/A	N/A	N/A	N/A
83	6-Aminocaproic Acid	132.095	69	131.1	C_6_H_13_NO_2_	3.76	N/A	N/A	N/A	N/A
84	5-Aminovaleric Acid	118.2	55	117.08	C_5_H_11_NO_2_	4.68	29,796.26	40,011.34	23,270.76	35,215.87
85	3,7-Dimethyluric Acid	195	180	196.06	C_7_H_8_N_4_O_3_	1.65	N/A	N/A	N/A	N/A
86	Creatine	132.1	90	131.07	C_4_H_9_N_3_O_2_	8.09	2174.93	4947.67	2376.90	2622.21
87	3-Hydroxyhippuric Acid	196.05	121	195.05	C_9_H_9_NO_4_	1.98	N/A	N/A	N/A	N/A
88	P-Aminohippuric Acid	195.069	120	194.07	C_9_H_10_N_2_O_3_	1.93	97.15	460.48	186.52	200.29
89	4-Acetamidobutyric Acid	146.074	86	145.07	C_6_H_11_NO_3_	1.17	1560.37	3167.75	1576.43	2300.27
90	γ-Aminobutyric Acid	104.06	68.8	103.06	C_4_H_9_NO_2_	6.56	3,866,168.03	3,234,742.41	2,366,972.33	484,156.29
91	kynurenine	209.1	146	208.09	C_10_H_12_N_2_O_3_	4.48	148.87	595.04	249.46	80.48
92	α-Aminoadipic acid	162.07	98.06	161.16	C_6_H_11_NO_4_	8.97	360,464.64	460,812.56	273,762.06	508,061.11
93	2-Aminoethanesulfonic Acid	124	80	125.15	C_2_H_7_NO_3_S	6.49	26,317.34	37,667.76	21,330.53	41,370.20
94	1,3,7-Trimethyluric Acid	211.08	154.06	210.08	C_8_H_10_N_4_O_3_	1.09	N/A	N/A	N/A	N/A

Note: Q1 refers to the molecular weight of a compound’s parent ion following its passage through an electrospray ion source, along with the addition of ions. Q3 denotes the specific fragment ion characteristic. RT stands for retention time, measured in minutes. N/A indicates non-detection. DG, YG, HG, and CK denote the average value within a specific group.

## Data Availability

All data used in this study can be found in manuscripts or supplementary materials and can also be obtained free of charge from correspondents.

## Supplementary Materials

The following supporting information can be downloaded at: https://www.mdpi.com/article/10.3390/metabo14080459/s1, Table S1: Detailed information of standard products; Table S2: SD and RSD values for four groups of samples; Table S3: Equations for quantitative determination of 94 amino acids metabolites; Table S4: Quantitative assay contents of 94 amino acids metabolite; Table S5: Names of 48 significantly different amino acids metabolite. Figure S1: TIC overlap chart. Figure S2: TIC plot for 12 samples; Figure S3: Violin plot of 48 differential metabolites. Figure S4–S6: Classification of metabolites using KEGG pathways.
