# Supplementary material for: Effects of Different Drying Methods on Amino Acid Metabolite Content and Quality of Ophiocordyceps sinensis by LC-MS/MS Combined with Multivariate Statistical Methods
Source: Metabolites. 2024 Aug 18;14(8):459. doi: 10.3390/metabo14080459 (PMC11356467; doi:10.3390/metabo14080459)
Supplement: Supplementary file 1 [file metabolites-14-00459-s001.zip › supplementary materials/FigureS4.pdf]

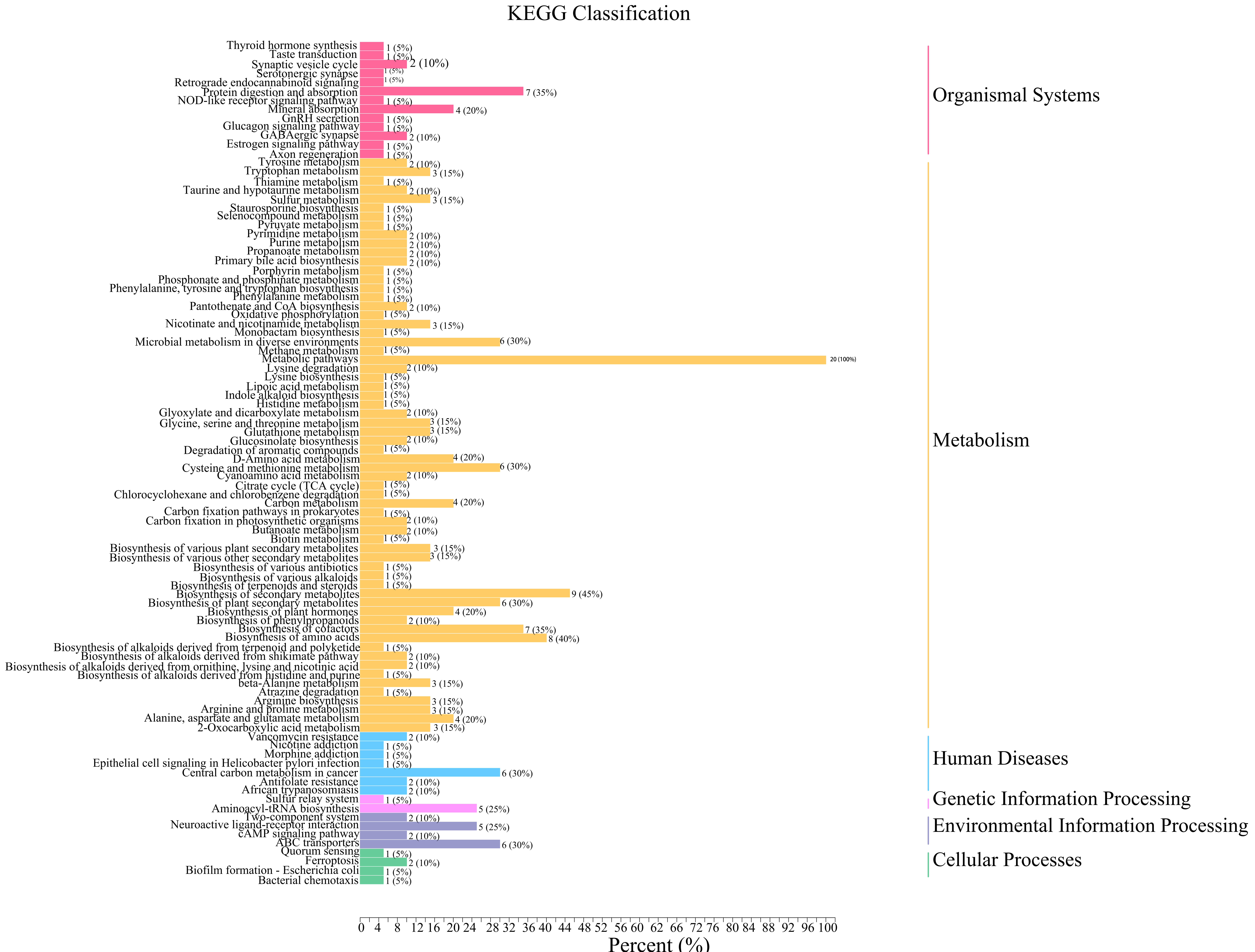
KEGG Classification
Thyroid hormone synthesis 1 (5%)
Taste transduction 1 (5%)
Synaptic vesicle cycle 2 (10%)
Serotonergic synapse 1 (5%)
Retrograde endocannabinoid signaling 1 (5%)
Protein digestion and absorption 7 (35%)
NOD-like receptor signaling pathway 1 (5%)
Mineral absorption 4 (20%)
GnRH secretion 1 (5%)
Glucagon signaling pathway 1 (5%)
GABAergic synapse 2 (10%)
Estrogen signaling pathway 1 (5%)
Axon regeneration 1 (5%)
Tyrosine metabolism 2 (10%)
Tryptophan metabolism 3 (15%)
Thiamine metabolism 1 (5%)
Taurine and hypotaurine metabolism 2 (10%)
Sulfur metabolism 3 (15%)
Staurosporine biosynthesis 1 (5%)
Selenocompound metabolism 1 (5%)
Pyruvate metabolism 1 (5%)
Pyrimidine metabolism 2 (10%)
Purine metabolism 2 (10%)
Propanoate metabolism 2 (10%)
Primary bile acid biosynthesis 2 (10%)
Porphyrin metabolism 1 (5%)
Phosphonate and phosphinate metabolism 1 (5%)
Phenylalanine, tyrosine and tryptophan biosynthesis 1 (5%)
Phenylalanine metabolism 1 (5%)
Pantothenate and CoA biosynthesis 2 (10%)
Oxidative phosphorylation 1 (5%)
Nicotinate and nicotinamide metabolism 3 (15%)
Monobactam biosynthesis 1 (5%)
Microbial metabolism in diverse environments 6 (30%)
Methane metabolism 1 (5%)
Metabolic pathways 20 (100%)
Lysine degradation 2 (10%)
Lysine biosynthesis 1 (5%)
Lipoic acid metabolism 1 (5%)
Indole alkaloid biosynthesis 1 (5%)
Histidine metabolism 1 (5%)
Glyoxylate and dicarboxylate metabolism 2 (10%)
Glycine, serine and threonine metabolism 3 (15%)
Glutathione metabolism 3 (15%)
Glucosinolate biosynthesis 2 (10%)
Degradation of aromatic compounds 1 (5%)
D-Amino acid metabolism 4 (20%)
Cysteine and methionine metabolism 6 (30%)
Cyanoamino acid metabolism 2 (10%)
Citrate cycle (TCA cycle) 1 (5%)
Chlorocyclohexane and chlorobenzene degradation 1 (5%)
Carbon metabolism 4 (20%)
Carbon fixation pathways in prokaryotes 1 (5%)
Carbon fixation in photosynthetic organisms 2 (10%)
Butanoate metabolism 2 (10%)
Biotin metabolism 1 (5%)
Biosynthesis of various plant secondary metabolites 3 (15%)
Biosynthesis of various other secondary metabolites 3 (15%)
Biosynthesis of various antibiotics 1 (5%)
Biosynthesis of various alkaloids 1 (5%)
Biosynthesis of terpenoids and steroids 1 (5%)
Biosynthesis of secondary metabolites 9 (45%)
Biosynthesis of plant secondary metabolites 6 (30%)
Biosynthesis of plant hormones 4 (20%)
Biosynthesis of phenylpropanoids 2 (10%)
Biosynthesis of cofactors 7 (35%)
Biosynthesis of amino acids 8 (40%)
Biosynthesis of alkaloids derived from terpenoid and polyketide 1 (5%)
Biosynthesis of alkaloids derived from shikimate pathway 2 (10%)
Biosynthesis of alkaloids derived from ornithine, lysine and nicotinic acid 2 (10%)
Biosynthesis of alkaloids derived from histidine and purine 1 (5%)
beta-Alanine metabolism 3 (15%)
Atrazine degradation 1 (5%)
Arginine biosynthesis 3 (15%)
Arginine and proline metabolism 3 (15%)
Alanine, aspartate and glutamate metabolism 4 (20%)
2-Oxocarboxylic acid metabolism 3 (15%)
Vancomycin resistance 2 (10%)
Nicotine addiction 1 (5%)
Morphine addiction 1 (5%)
Epithelial cell signaling in Helicobacter pylori infection 1 (5%)
Central carbon metabolism in cancer 6 (30%)
Antifolate resistance 2 (10%)
African trypanosomiasis 2 (10%)
Sulfur relay system 1 (5%)
Aminoacyl-tRNA biosynthesis 5 (25%)
Two-component system 2 (10%)
Neuroactive ligand-receptor interaction 5 (25%)
cAMP signaling pathway 2 (10%)
ABC transporters 6 (30%)
Quorum sensing 1 (5%)
Ferroptosis 2 (10%)
Biofilm formation - Escherichia coli 1 (5%)
Bacterial chemotaxis 1 (5%)
Organismal Systems
Metabolism
Human Diseases
Genetic Information Processing
Environmental Information Processing
Cellular Processes
0 4 8 12 16 20 24 28 32 36 40 44 48 52 56 60 62 66 72 76 80 84 88 92 96 100
Percent (%)
