# Supplementary material for: Effects of Different Drying Methods on Amino Acid Metabolite Content and Quality of Ophiocordyceps sinensis by LC-MS/MS Combined with Multivariate Statistical Methods
Source: Metabolites. 2024 Aug 18;14(8):459. doi: 10.3390/metabo14080459 (PMC11356467; doi:10.3390/metabo14080459)
Supplement: Supplementary file 1 [file metabolites-14-00459-s001.zip › supplementary materials/FigureS5.pdf]

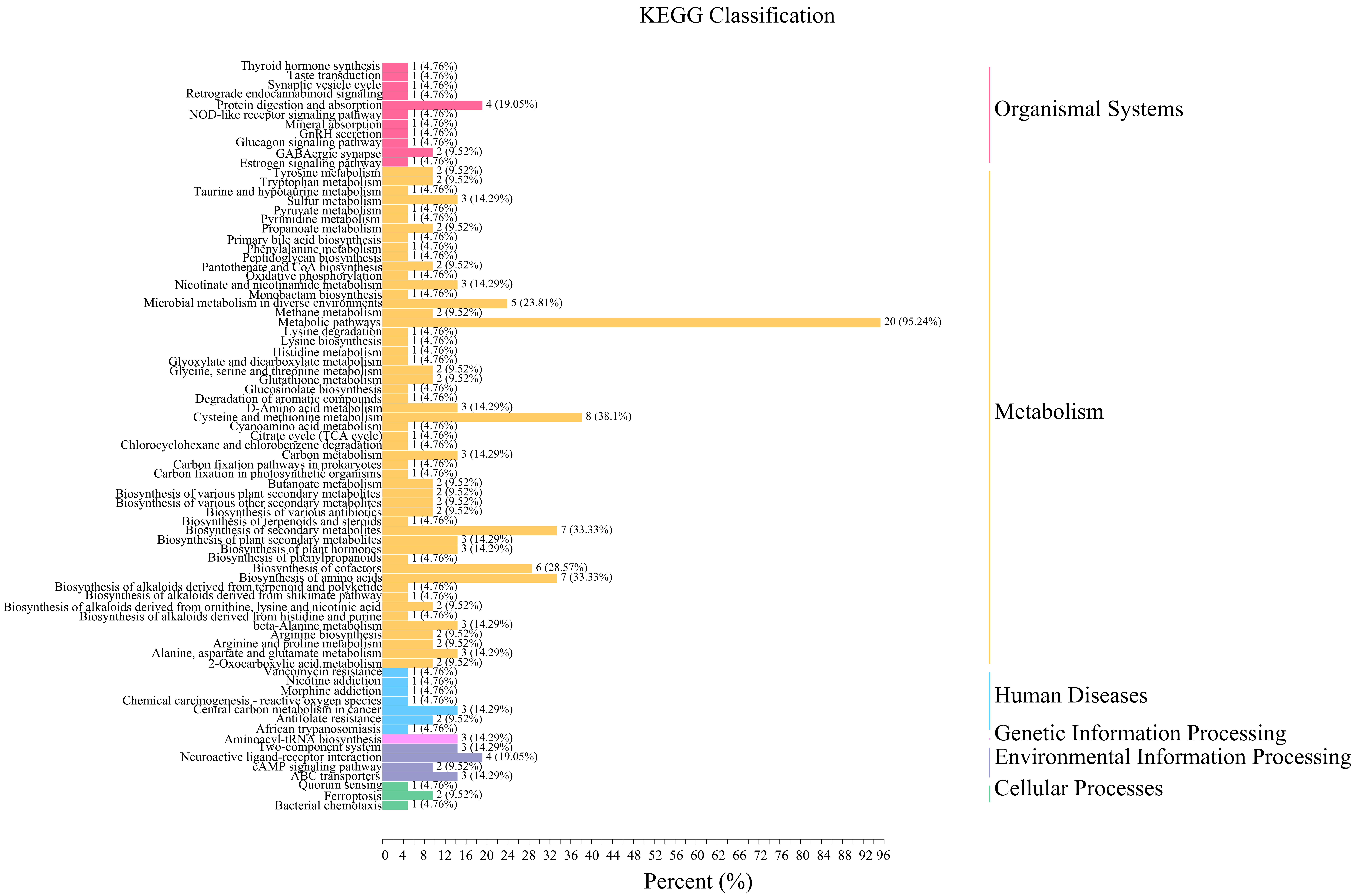
KEGG Classification
Thyroid hormone synthesis 1 (4.76%)
Taste transduction 1 (4.76%)
Synaptic vesicle cycle 1 (4.76%)
Retrograde endocannabinoid signaling 1 (4.76%)
Protein digestion and absorption 4 (19.05%)
NOD-like receptor signaling pathway 1 (4.76%)
Mineral absorption 1 (4.76%)
GnRH secretion 1 (4.76%)
Glucagon signaling pathway 1 (4.76%)
GABAergic synapse 2 (9.52%)
Estrogen signaling pathway 1 (4.76%)
Tyrosine metabolism 2 (9.52%)
Tryptophan metabolism 2 (9.52%)
Taurine and hypotaurine metabolism 1 (4.76%)
Sulfur metabolism 3 (14.29%)
Pyruvate metabolism 1 (4.76%)
Pyrimidine metabolism 1 (4.76%)
Propanoate metabolism 2 (9.52%)
Primary bile acid biosynthesis 1 (4.76%)
Phenylalanine metabolism 1 (4.76%)
Peptidoglycan biosynthesis 1 (4.76%)
Pantothenate and CoA biosynthesis 2 (9.52%)
Oxidative phosphorylation 1 (4.76%)
Nicotinate and nicotinamide metabolism 3 (14.29%)
Monobactam biosynthesis 1 (4.76%)
Microbial metabolism in diverse environments 5 (23.81%)
Methane metabolism 2 (9.52%)
Metabolic pathways 20 (95.24%)
Lysine degradation 1 (4.76%)
Lysine biosynthesis 1 (4.76%)
Histidine metabolism 1 (4.76%)
Glyoxylate and dicarboxylate metabolism 1 (4.76%)
Glycine, serine and threonine metabolism 2 (9.52%)
Glutathione metabolism 2 (9.52%)
Glucosinolate biosynthesis 1 (4.76%)
Degradation of aromatic compounds 1 (4.76%)
D-Amino acid metabolism 3 (14.29%)
Cysteine and methionine metabolism 8 (38.1%)
Cyanoamino acid metabolism 1 (4.76%)
Citrate cycle (TCA cycle) 1 (4.76%)
Chlorocyclohexane and chlorobenzene degradation 1 (4.76%)
Carbon metabolism 3 (14.29%)
Carbon fixation pathways in prokaryotes 1 (4.76%)
Carbon fixation in photosynthetic organisms 1 (4.76%)
Butanoate metabolism 2 (9.52%)
Biosynthesis of various plant secondary metabolites 2 (9.52%)
Biosynthesis of various other secondary metabolites 2 (9.52%)
Biosynthesis of various antibiotics 2 (9.52%)
Biosynthesis of terpenoids and steroids 1 (4.76%)
Biosynthesis of secondary metabolites 7 (33.33%)
Biosynthesis of plant secondary metabolites 3 (14.29%)
Biosynthesis of plant hormones 3 (14.29%)
Biosynthesis of phenylpropanoids 1 (4.76%)
Biosynthesis of cofactors 6 (28.57%)
Biosynthesis of amino acids 7 (33.33%)
Biosynthesis of alkaloids derived from terpenoid and polyketide 1 (4.76%)
Biosynthesis of alkaloids derived from shikimate pathway 1 (4.76%)
Biosynthesis of alkaloids derived from ornithine, lysine and nicotinic acid 2 (9.52%)
Biosynthesis of alkaloids derived from histidine and purine 1 (4.76%)
beta-Alanine metabolism 3 (14.29%)
Arginine biosynthesis 2 (9.52%)
Arginine and proline metabolism 2 (9.52%)
Alanine, aspartate and glutamate metabolism 3 (14.29%)
2-Oxocarboxylic acid metabolism 2 (9.52%)
Vancomycin resistance 1 (4.76%)
Nicotine addiction 1 (4.76%)
Morphine addiction 1 (4.76%)
Chemical carcinogenesis - reactive oxygen species 1 (4.76%)
Central carbon metabolism in cancer 3 (14.29%)
Antifolate resistance 2 (9.52%)
African trypanosomiasis 1 (4.76%)
Aminoacyl-tRNA biosynthesis 3 (14.29%)
Two-component system 3 (14.29%)
Neuroactive ligand-receptor interaction 4 (19.05%)
cAMP signaling pathway 2 (9.52%)
ABC transporters 3 (14.29%)
Quorum sensing 1 (4.76%)
Ferroptosis 2 (9.52%)
Bacterial chemotaxis 1 (4.76%)
Organismal Systems
Metabolism
Human Diseases
Genetic Information Processing
Environmental Information Processing
Cellular Processes
0 4 8 12 16 20 24 28 32 36 40 44 48 52 56 60 62 66 72 76 80 84 88 92 96
Percent (%)
