# Supplementary material for: Effects of Different Drying Methods on Amino Acid Metabolite Content and Quality of Ophiocordyceps sinensis by LC-MS/MS Combined with Multivariate Statistical Methods
Source: Metabolites. 2024 Aug 18;14(8):459. doi: 10.3390/metabo14080459 (PMC11356467; doi:10.3390/metabo14080459)
Supplement: Supplementary file 1 [file metabolites-14-00459-s001.zip › supplementary materials/FigureS6.pdf]

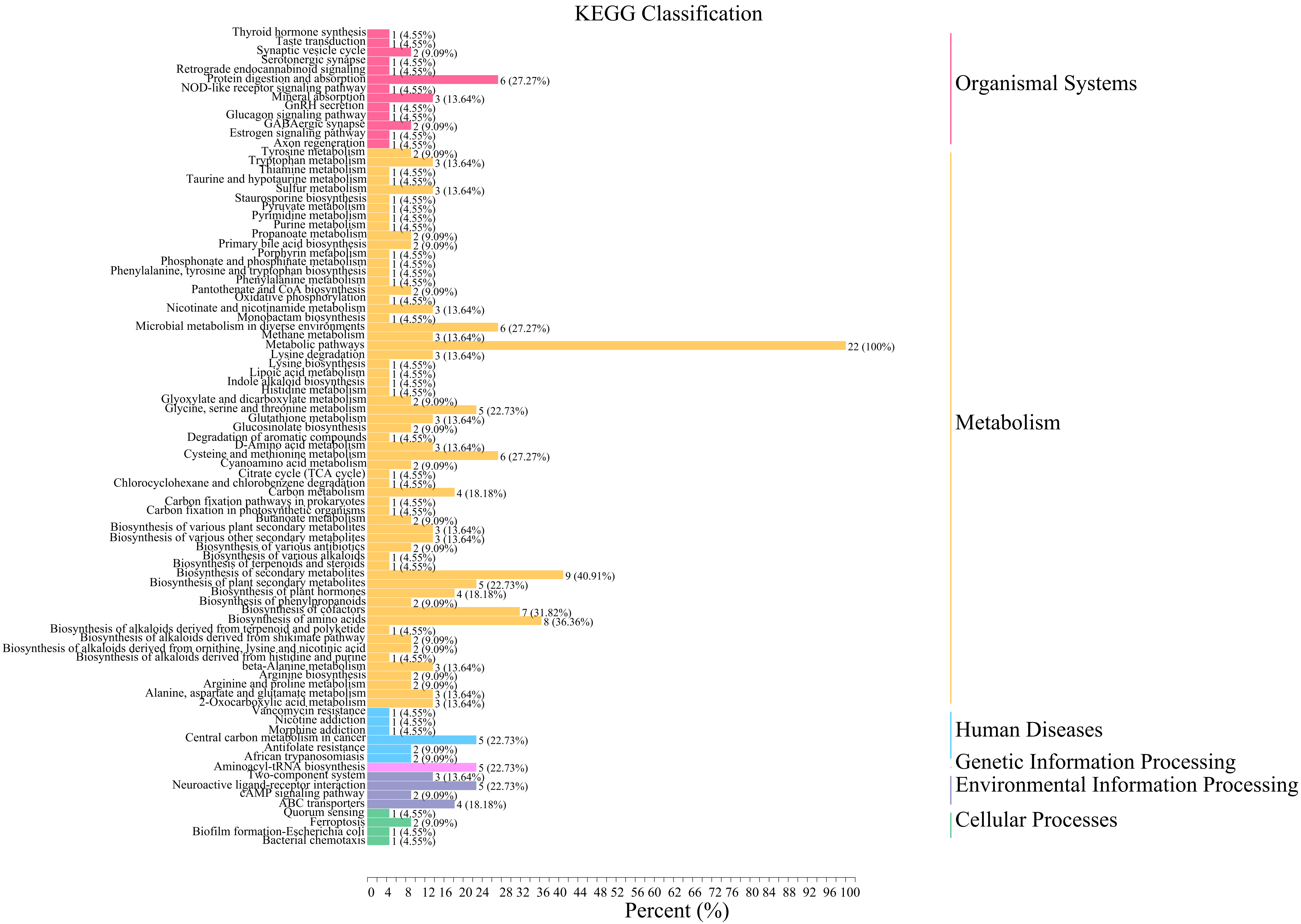

KEGG Classification
Thyroid hormone synthesis 1 (4.55%)
Taste transduction 1 (4.55%)
Synaptic vesicle cycle 2 (9.09%)
Serotonergic synapse 1 (4.55%)
Retrograde endocannabinoid signaling 1 (4.55%)
Protein digestion and absorption 6 (27.27%)
NOD-like receptor signaling pathway 1 (4.55%)
Mineral absorption 3 (13.64%)
GnRH secretion 1 (4.55%)
Glucagon signaling pathway 1 (4.55%)
GABAergic synapse 2 (9.09%)
Estrogen signaling pathway 1 (4.55%)
Axon regeneration 1 (4.55%)
Tyrosine metabolism 2 (9.09%)
Tryptophan metabolism 3 (13.64%)
Thiamine metabolism 1 (4.55%)
Taurine and hypotaurine metabolism 1 (4.55%)
Sulfur metabolism 3 (13.64%)
Staurosporine biosynthesis 1 (4.55%)
Pyruvate metabolism 1 (4.55%)
Pyrimidine metabolism 1 (4.55%)
Purine metabolism 1 (4.55%)
Propanoate metabolism 2 (9.09%)
Primary bile acid biosynthesis 2 (9.09%)
Porphyrin metabolism 1 (4.55%)
Phosphonate and phosphinate metabolism 1 (4.55%)
Phenylalanine, tyrosine and tryptophan biosynthesis 1 (4.55%)
Phenylalanine metabolism 1 (4.55%)
Pantothenate and CoA biosynthesis 2 (9.09%)
Oxidative phosphorylation 1 (4.55%)
Nicotinate and nicotinamide metabolism 3 (13.64%)
Monobactam biosynthesis 1 (4.55%)
Microbial metabolism in diverse environments 6 (27.27%)
Methane metabolism 3 (13.64%)
Metabolic pathways 22 (100%)
Lysine degradation 3 (13.64%)
Lysine biosynthesis 1 (4.55%)
Lipoic acid metabolism 1 (4.55%)
Indole alkaloid biosynthesis 1 (4.55%)
Histidine metabolism 1 (4.55%)
Glyoxylate and dicarboxylate metabolism 2 (9.09%)
Glycine, serine and threonine metabolism 5 (22.73%)
Glutathione metabolism 3 (13.64%)
Glucosinolate biosynthesis 2 (9.09%)
Degradation of aromatic compounds 1 (4.55%)
D-Amino acid metabolism 3 (13.64%)
Cysteine and methionine metabolism 6 (27.27%)
Cyanoamino acid metabolism 2 (9.09%)
Citrate cycle (TCA cycle) 1 (4.55%)
Chlorocyclohexane and chlorobenzene degradation 1 (4.55%)
Carbon metabolism 4 (18.18%)
Carbon fixation pathways in prokaryotes 1 (4.55%)
Carbon fixation in photosynthetic organisms 1 (4.55%)
Butanoate metabolism 2 (9.09%)
Biosynthesis of various plant secondary metabolites 3 (13.64%)
Biosynthesis of various other secondary metabolites 3 (13.64%)
Biosynthesis of various antibiotics 2 (9.09%)
Biosynthesis of various alkaloids 1 (4.55%)
Biosynthesis of terpenoids and steroids 1 (4.55%)
Biosynthesis of secondary metabolites 9 (40.91%)
Biosynthesis of plant secondary metabolites 5 (22.73%)
Biosynthesis of plant hormones 4 (18.18%)
Biosynthesis of phenylpropanoids 2 (9.09%)
Biosynthesis of cofactors 7 (31.82%)
Biosynthesis of amino acids 8 (36.36%)
Biosynthesis of alkaloids derived from terpenoid and polyketide 1 (4.55%)
Biosynthesis of alkaloids derived from shikimate pathway 2 (9.09%)
Biosynthesis of alkaloids derived from ornithine, lysine and nicotinic acid 2 (9.09%)
Biosynthesis of alkaloids derived from histidine and purine 1 (4.55%)
beta-Alanine metabolism 3 (13.64%)
Arginine biosynthesis 2 (9.09%)
Arginine and proline metabolism 2 (9.09%)
Alanine, aspartate and glutamate metabolism 3 (13.64%)
2-Oxocarboxylic acid metabolism 3 (13.64%)
Vancomycin resistance 1 (4.55%)
Nicotine addiction 1 (4.55%)
Morphine addiction 1 (4.55%)
Central carbon metabolism in cancer 5 (22.73%)
Antifolate resistance 2 (9.09%)
African trypanosomiasis 2 (9.09%)
Aminoacyl-tRNA biosynthesis 5 (22.73%)
Two-component system 3 (13.64%)
Neuroactive ligand-receptor interaction 5 (22.73%)
cAMP signaling pathway 2 (9.09%)
ABC transporters 4 (18.18%)
Quorum sensing 1 (4.55%)
Ferroptosis 2 (9.09%)
Biofilm formation-Escherichia coli 1 (4.55%)
Bacterial chemotaxis 1 (4.55%)
Organismal Systems
Metabolism
Human Diseases
Genetic Information Processing
Environmental Information Processing
Cellular Processes
0 4 8 12 16 20 24 28 32 36 40 44 48 52 56 60 62 66 72 76 80 84 88 92 96 100
Percent (%)
