# Supplementary figures and images for: Effects of Different Drying Methods on Amino Acid Metabolite Content and Quality of Ophiocordyceps sinensis by LC-MS/MS Combined with Multivariate Statistical Methods
Source: Metabolites. 2024 Aug 18;14(8):459. doi: 10.3390/metabo14080459 (PMC11356467; doi:10.3390/metabo14080459)

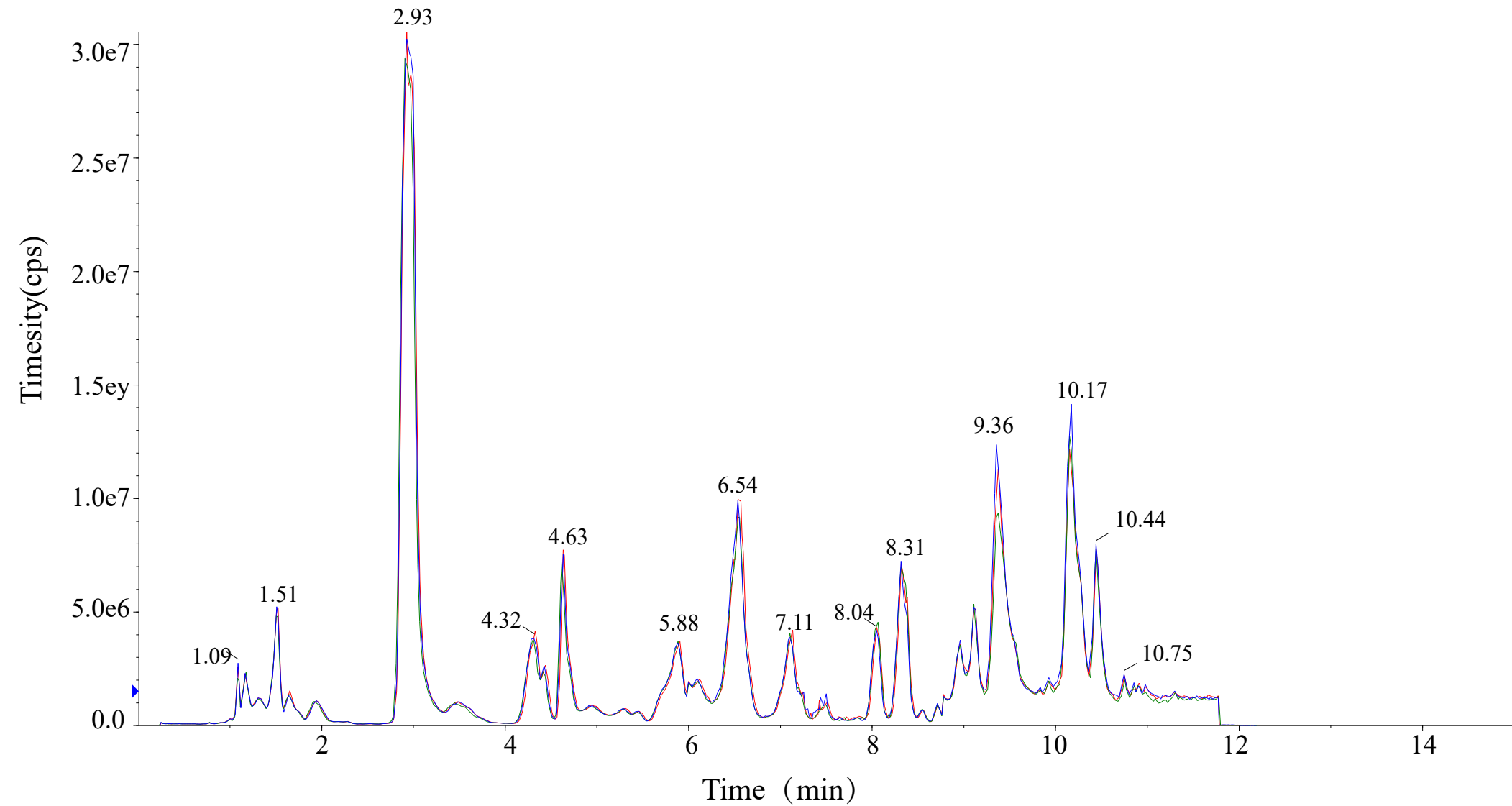

Supplement: Supplementary file 1 [file metabolites-14-00459-s001.zip › supplementary materials/Figure S1.pdf]

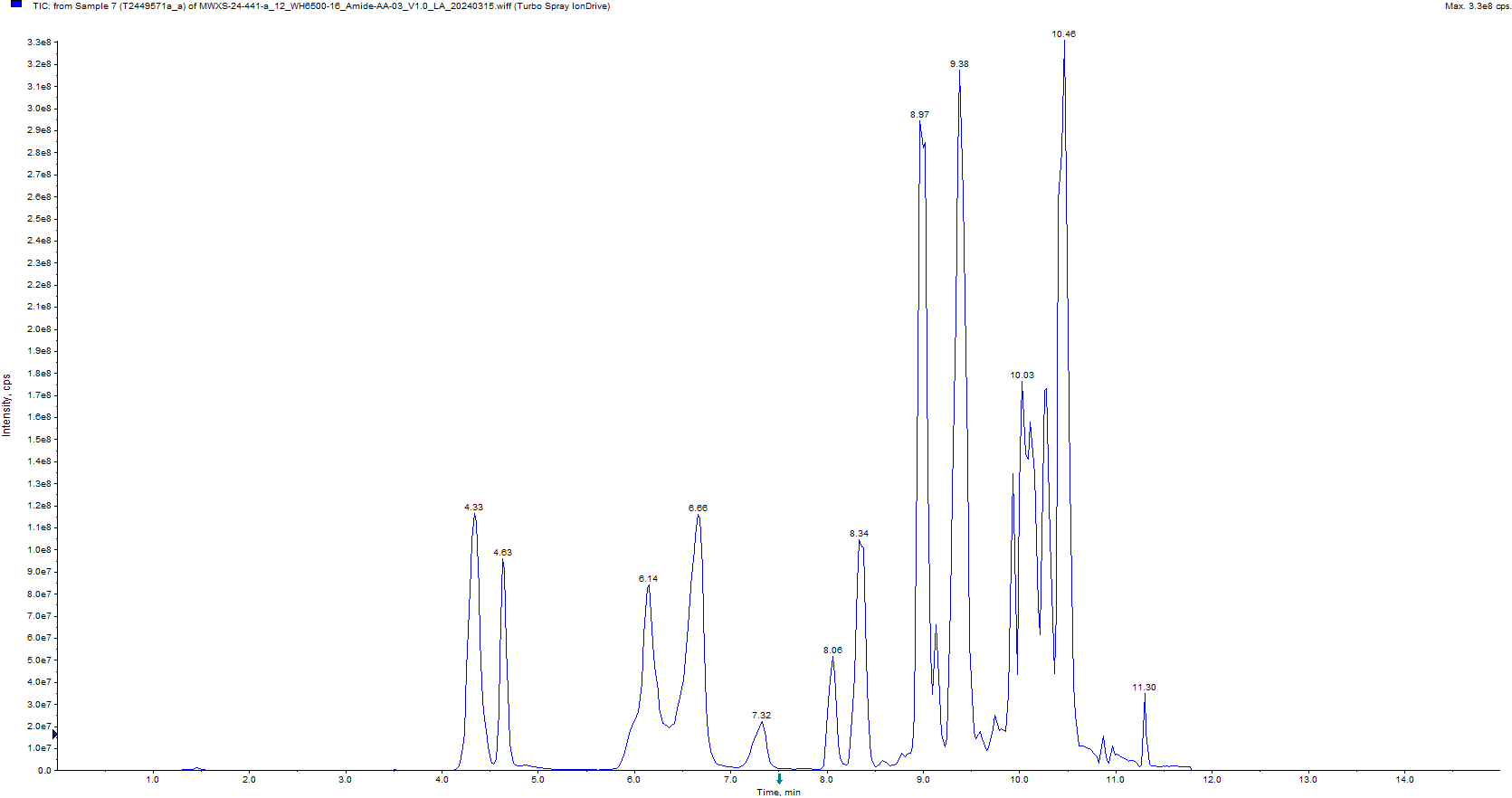

Supplement: Supplementary file 1 [file metabolites-14-00459-s001.zip › supplementary materials/Figure S2/MWXS-24-441-a_T2449571a_a_MS_TIC.png]

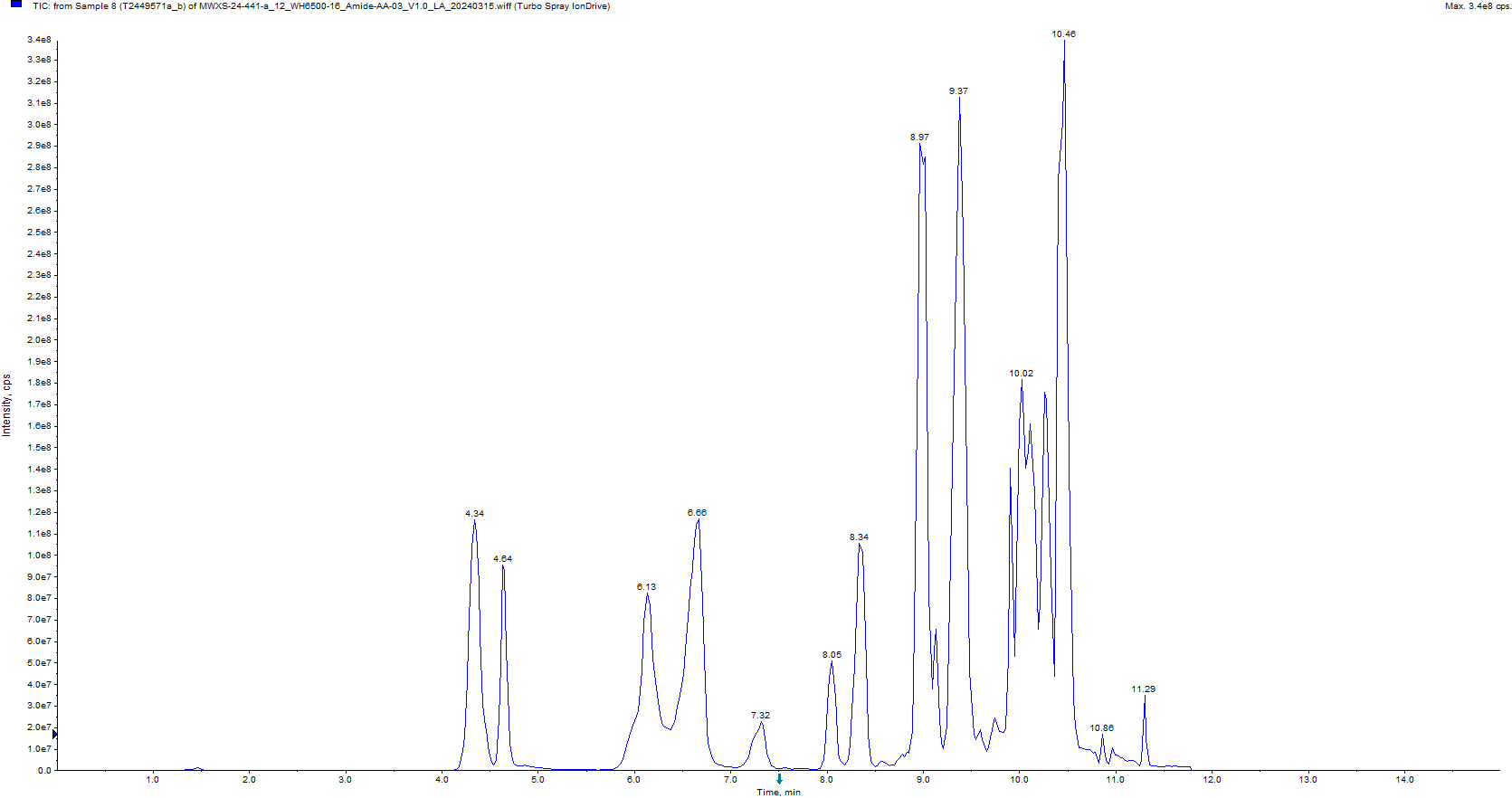

Supplement: Supplementary file 1 [file metabolites-14-00459-s001.zip › supplementary materials/Figure S2/MWXS-24-441-a_T2449571a_b_MS_TIC.png]

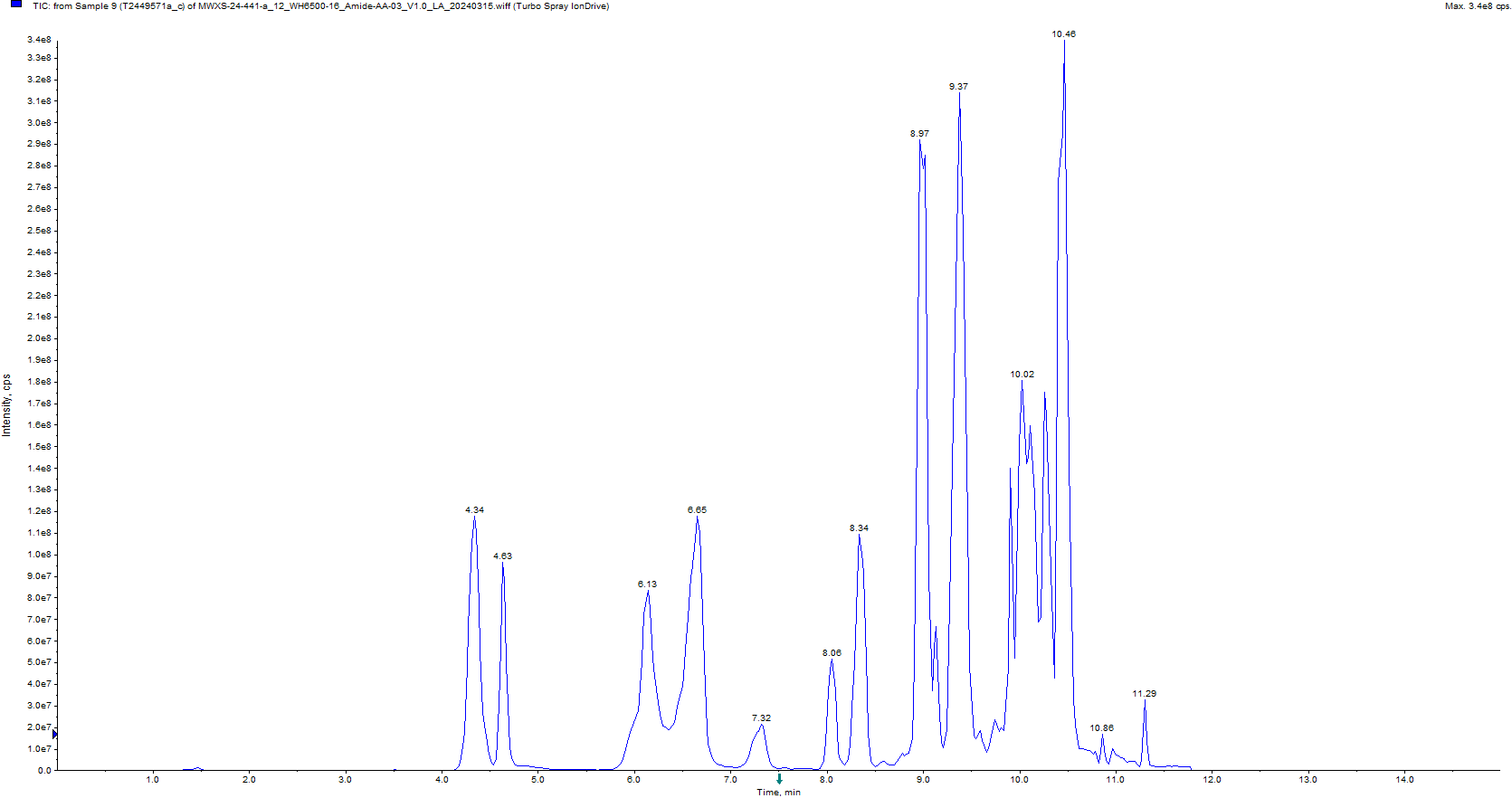

Supplement: Supplementary file 1 [file metabolites-14-00459-s001.zip › supplementary materials/Figure S2/MWXS-24-441-a_T2449571a_c_MS_TIC.png]

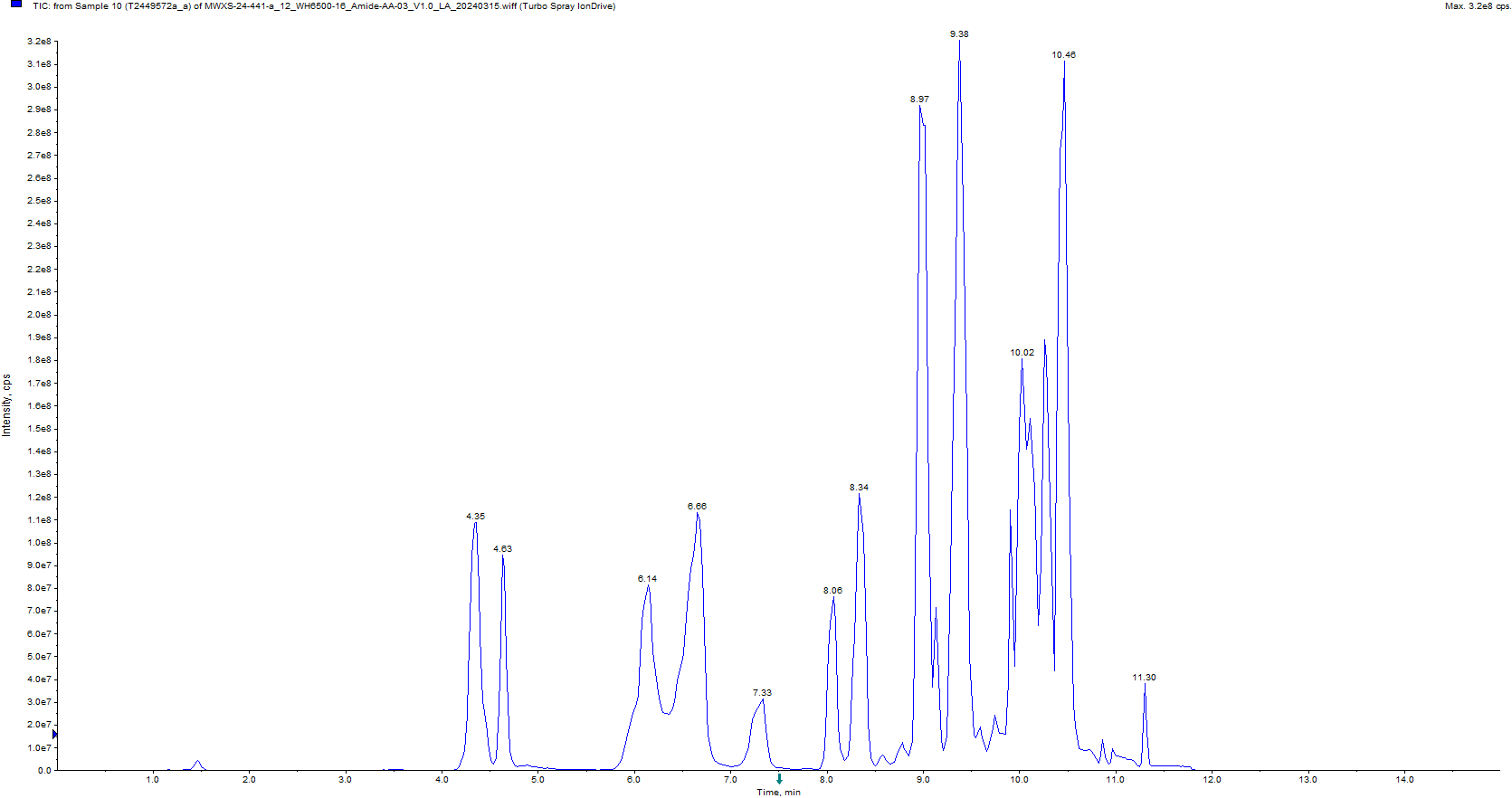

Supplement: Supplementary file 1 [file metabolites-14-00459-s001.zip › supplementary materials/Figure S2/MWXS-24-441-a_T2449572a_a_MS_TIC.png]

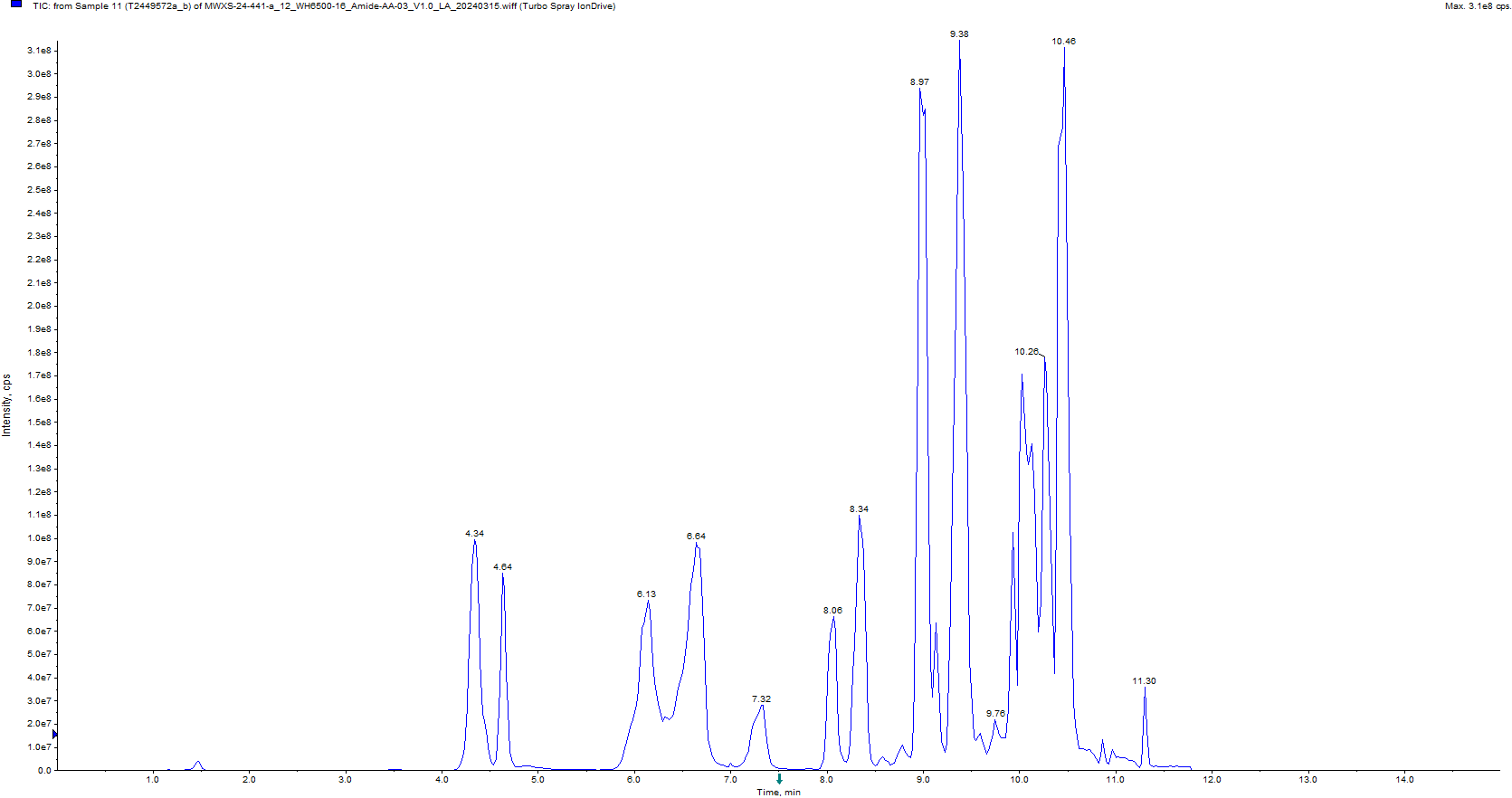

Supplement: Supplementary file 1 [file metabolites-14-00459-s001.zip › supplementary materials/Figure S2/MWXS-24-441-a_T2449572a_b_MS_TIC.png]

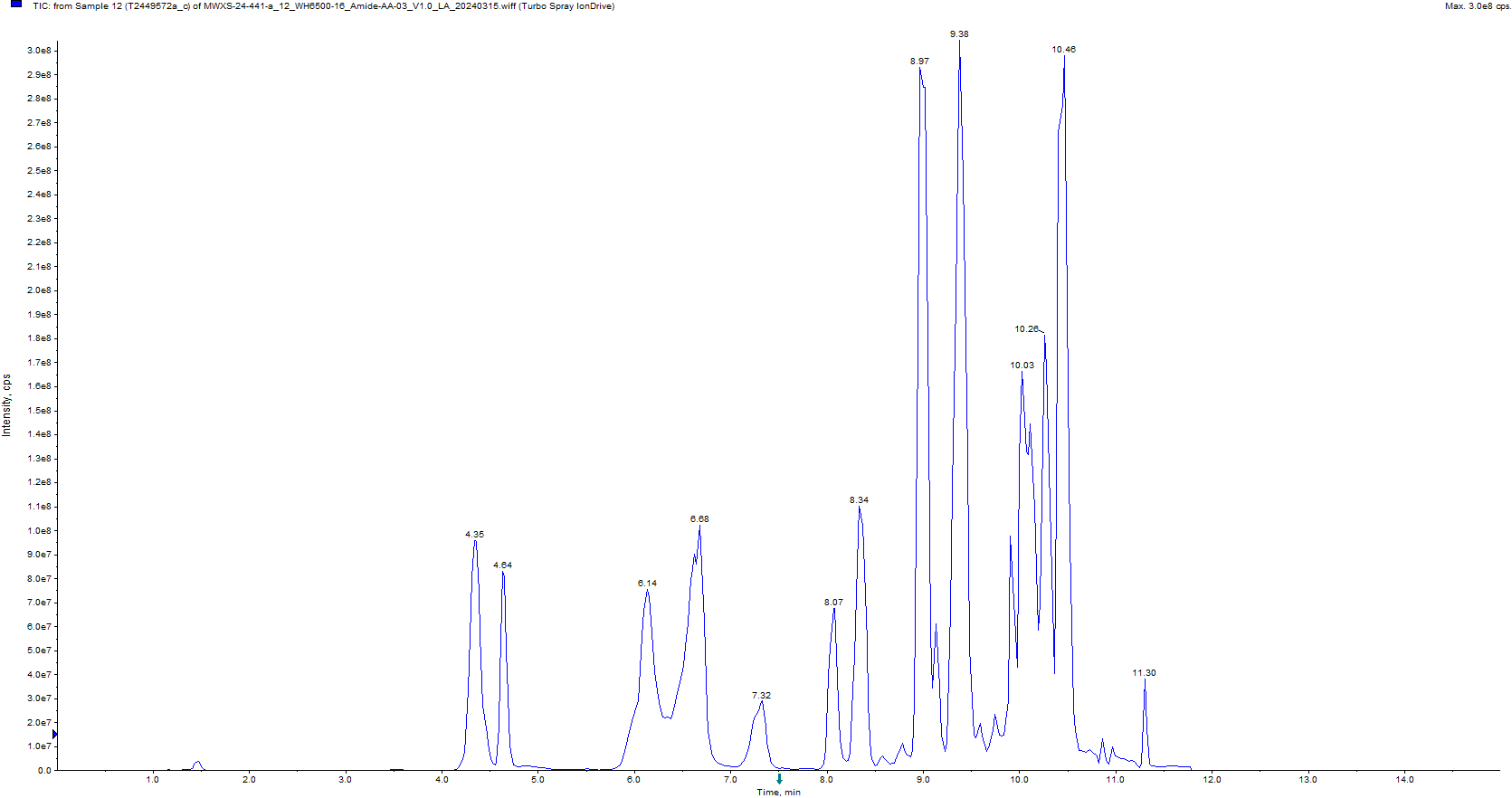

Supplement: Supplementary file 1 [file metabolites-14-00459-s001.zip › supplementary materials/Figure S2/MWXS-24-441-a_T2449572a_c_MS_TIC.png]

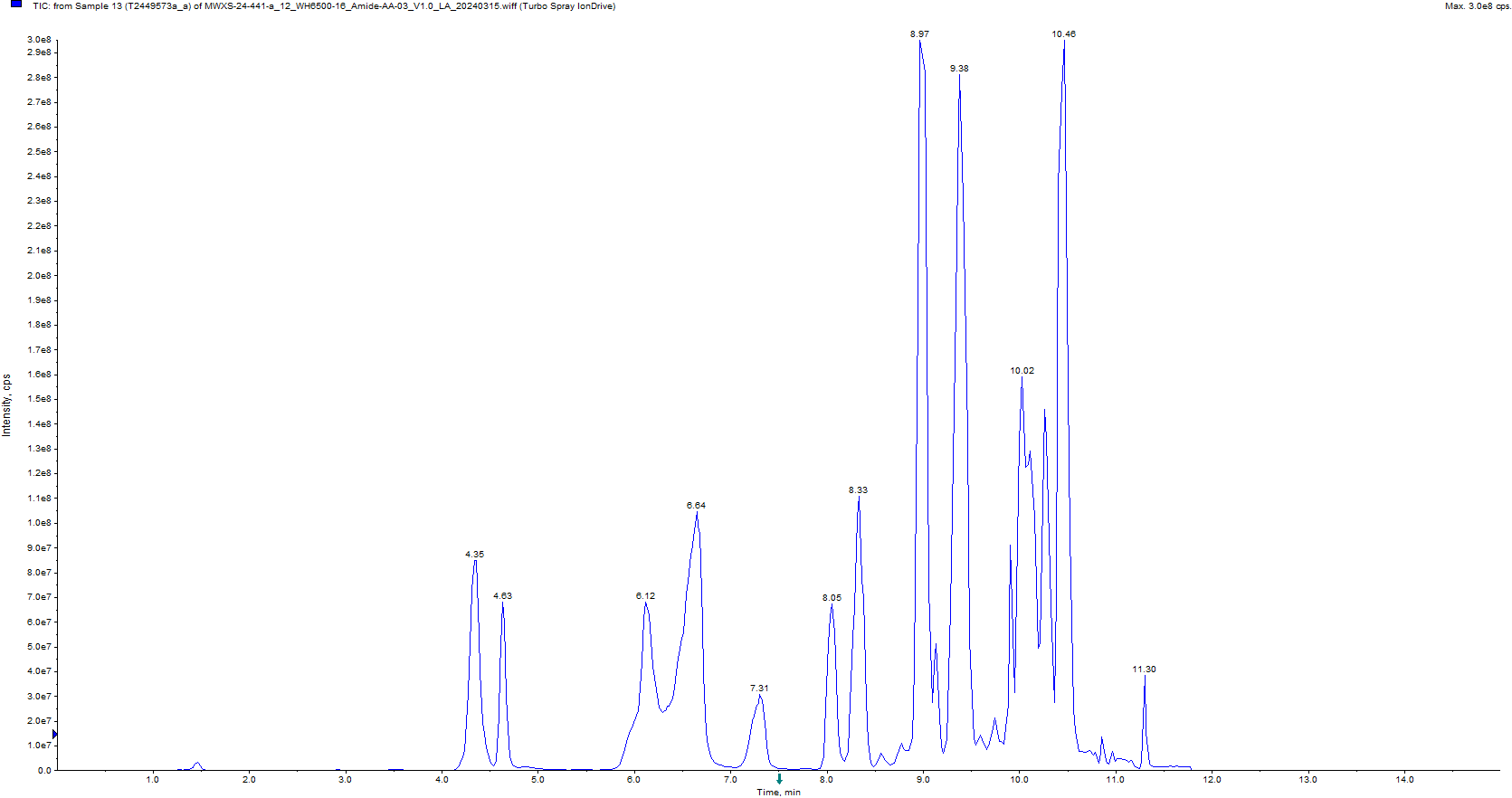

Supplement: Supplementary file 1 [file metabolites-14-00459-s001.zip › supplementary materials/Figure S2/MWXS-24-441-a_T2449573a_a_MS_TIC.png]

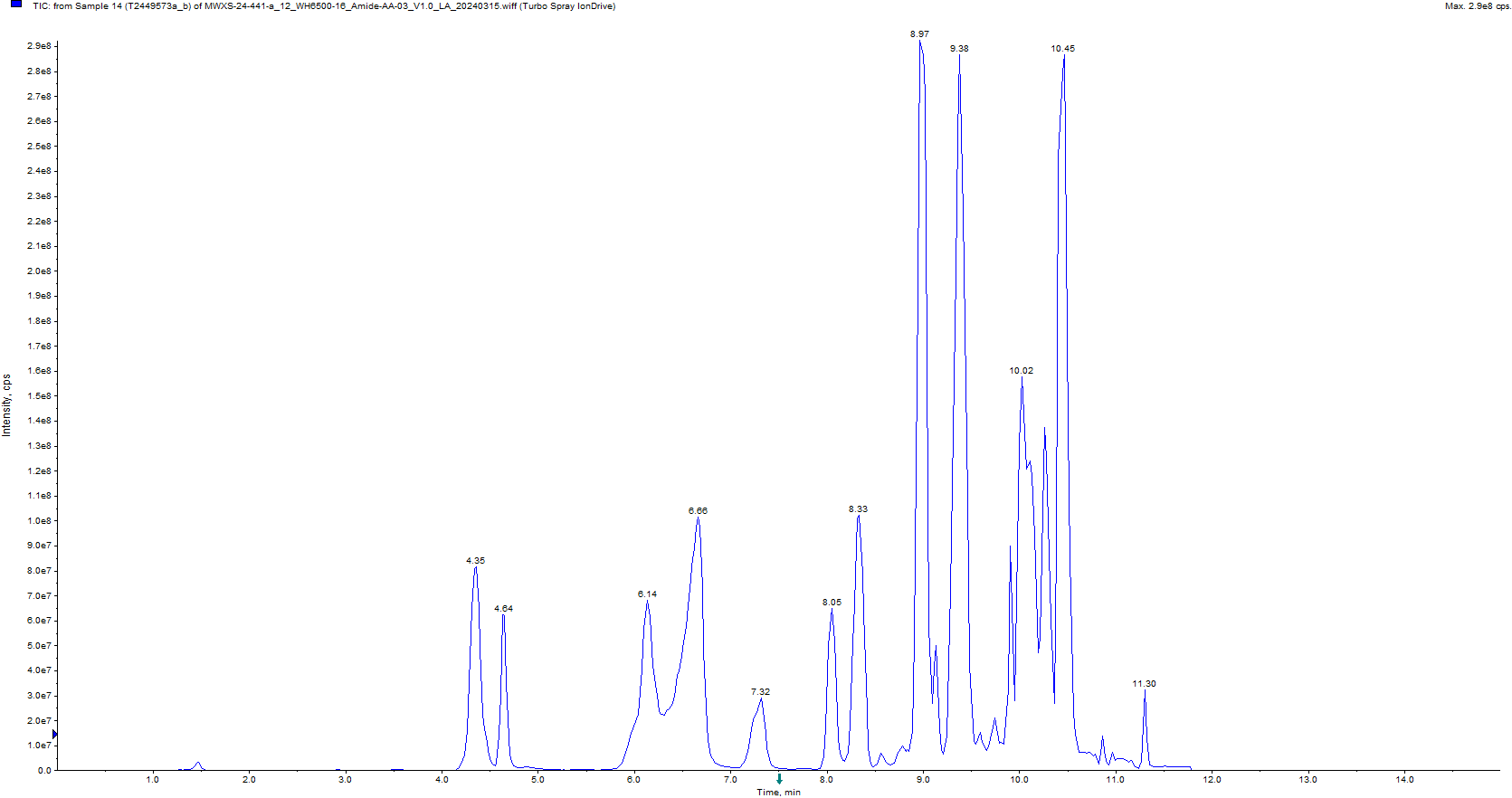

Supplement: Supplementary file 1 [file metabolites-14-00459-s001.zip › supplementary materials/Figure S2/MWXS-24-441-a_T2449573a_b_MS_TIC.png]

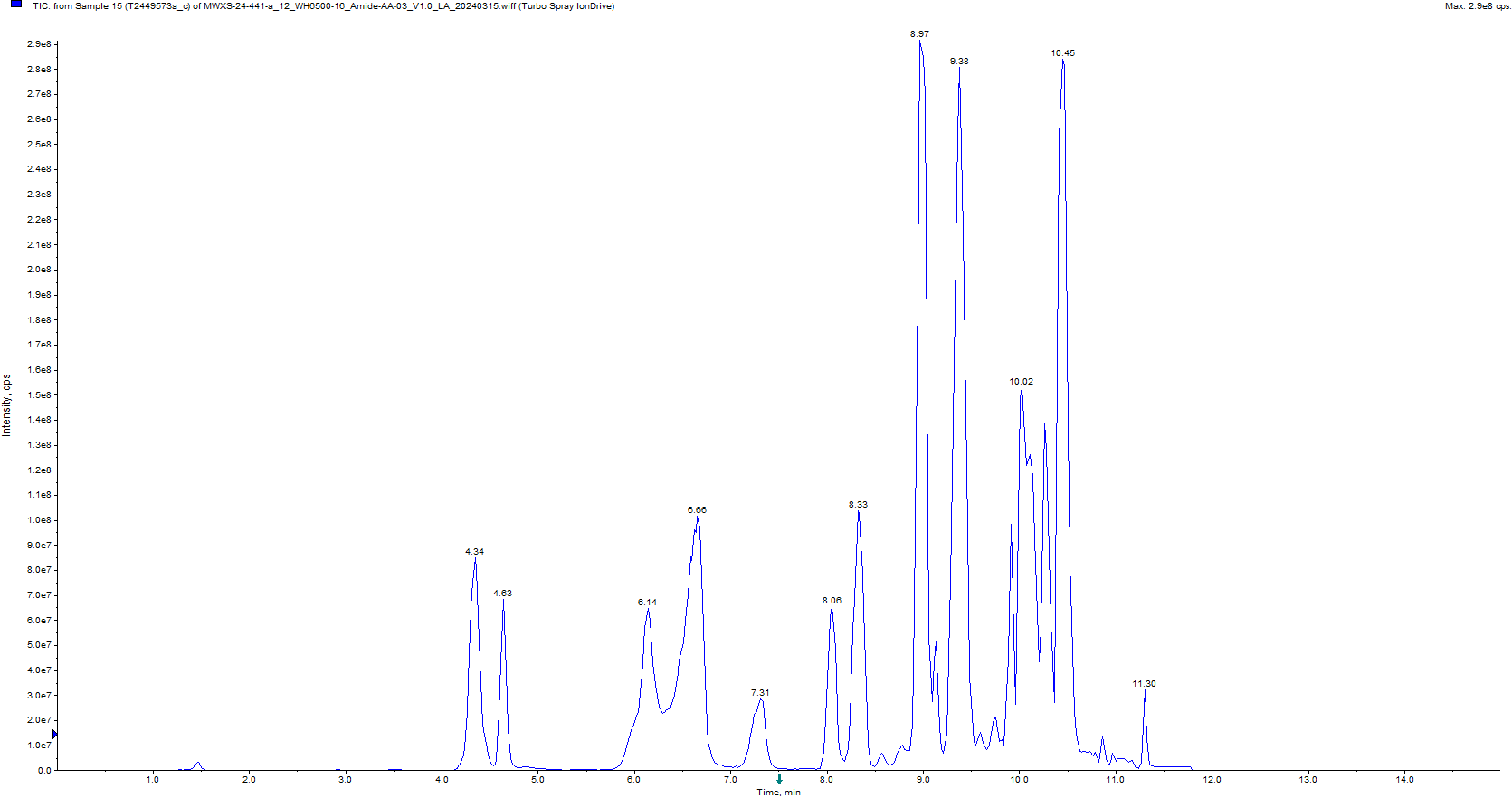

Supplement: Supplementary file 1 [file metabolites-14-00459-s001.zip › supplementary materials/Figure S2/MWXS-24-441-a_T2449573a_c_MS_TIC.png]

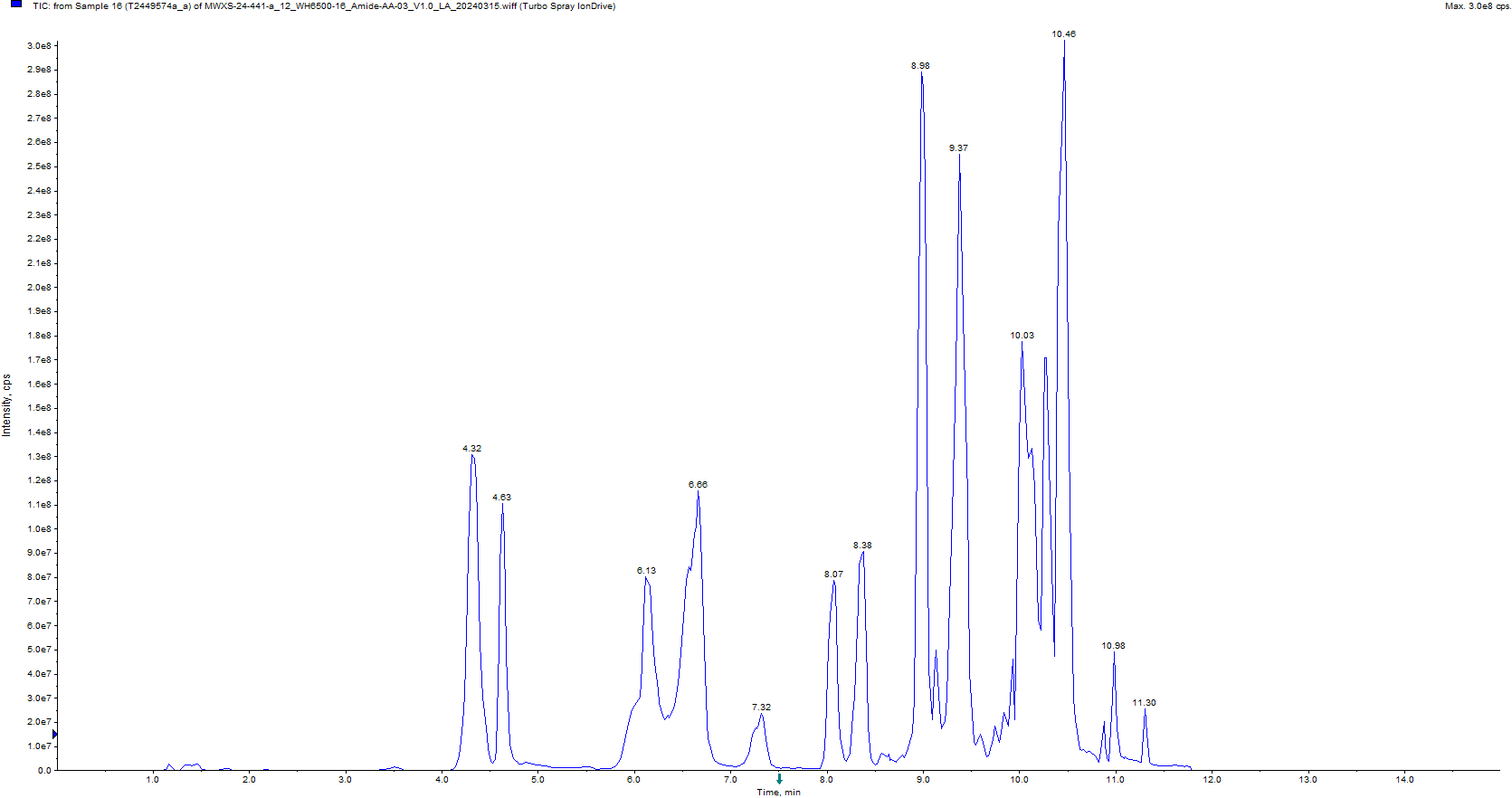

Supplement: Supplementary file 1 [file metabolites-14-00459-s001.zip › supplementary materials/Figure S2/MWXS-24-441-a_T2449574a_a_MS_TIC.png]

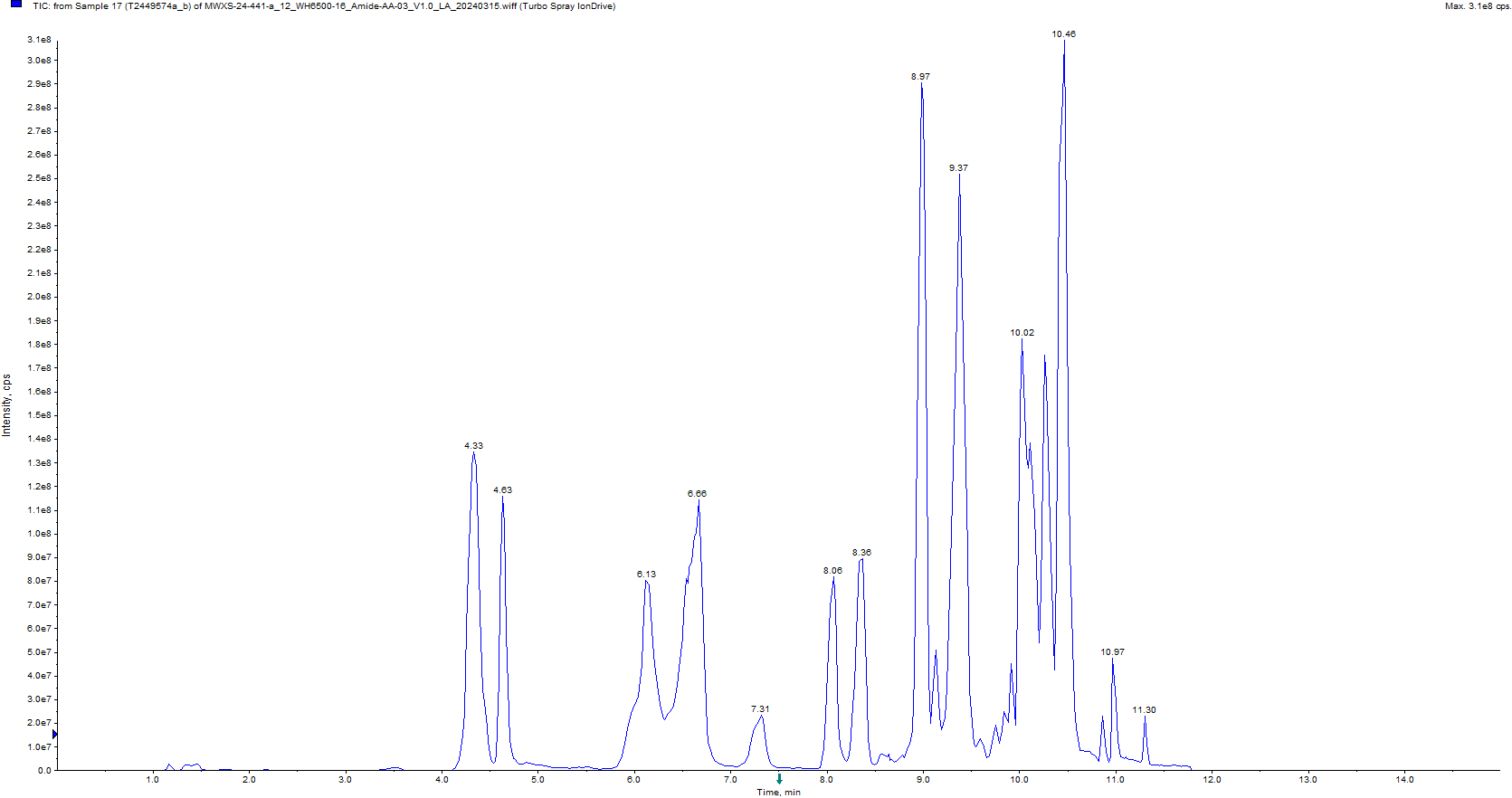

Supplement: Supplementary file 1 [file metabolites-14-00459-s001.zip › supplementary materials/Figure S2/MWXS-24-441-a_T2449574a_b_MS_TIC.png]

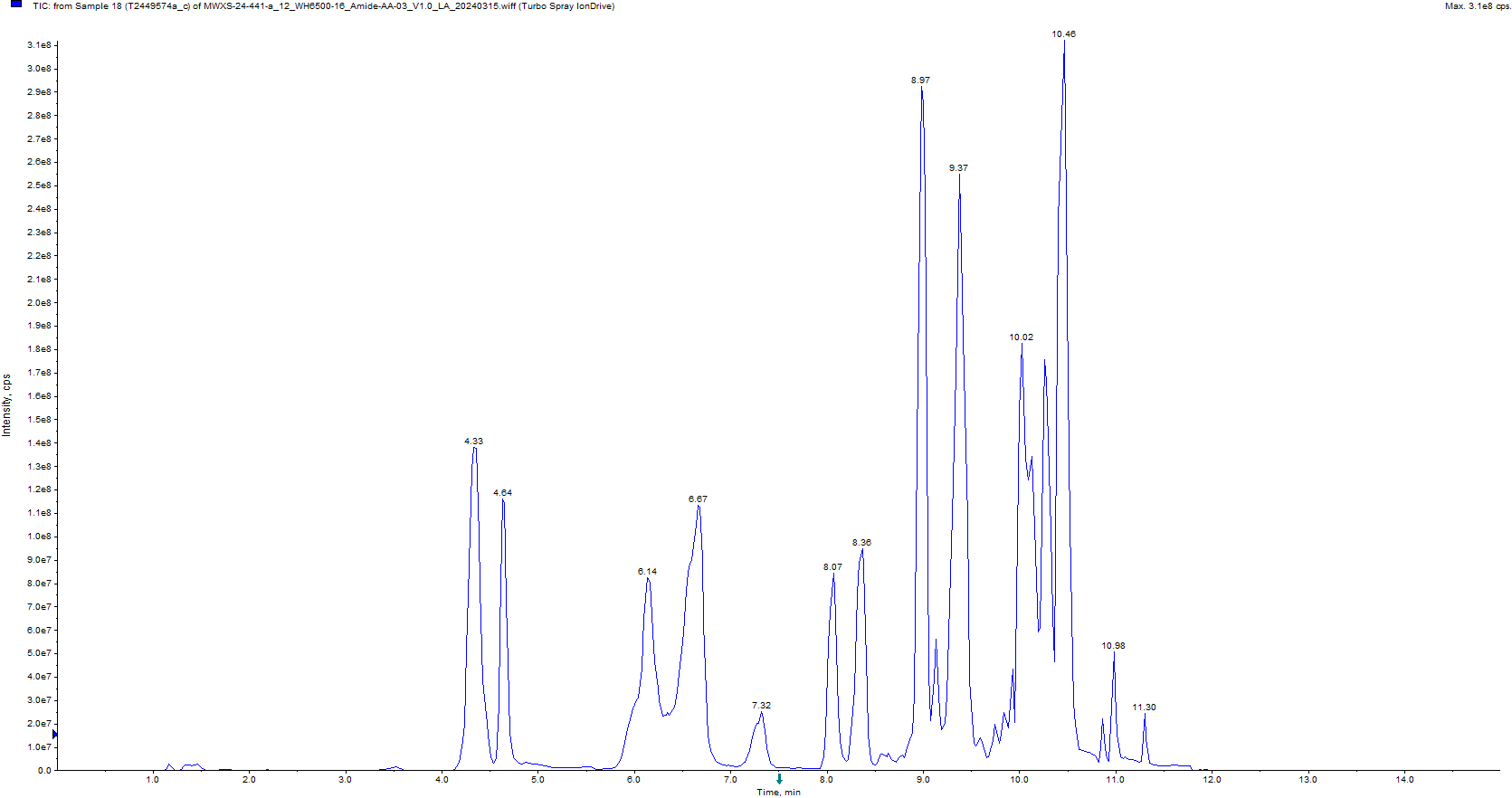

Supplement: Supplementary file 1 [file metabolites-14-00459-s001.zip › supplementary materials/Figure S2/MWXS-24-441-a_T2449574a_c_MS_TIC.png]

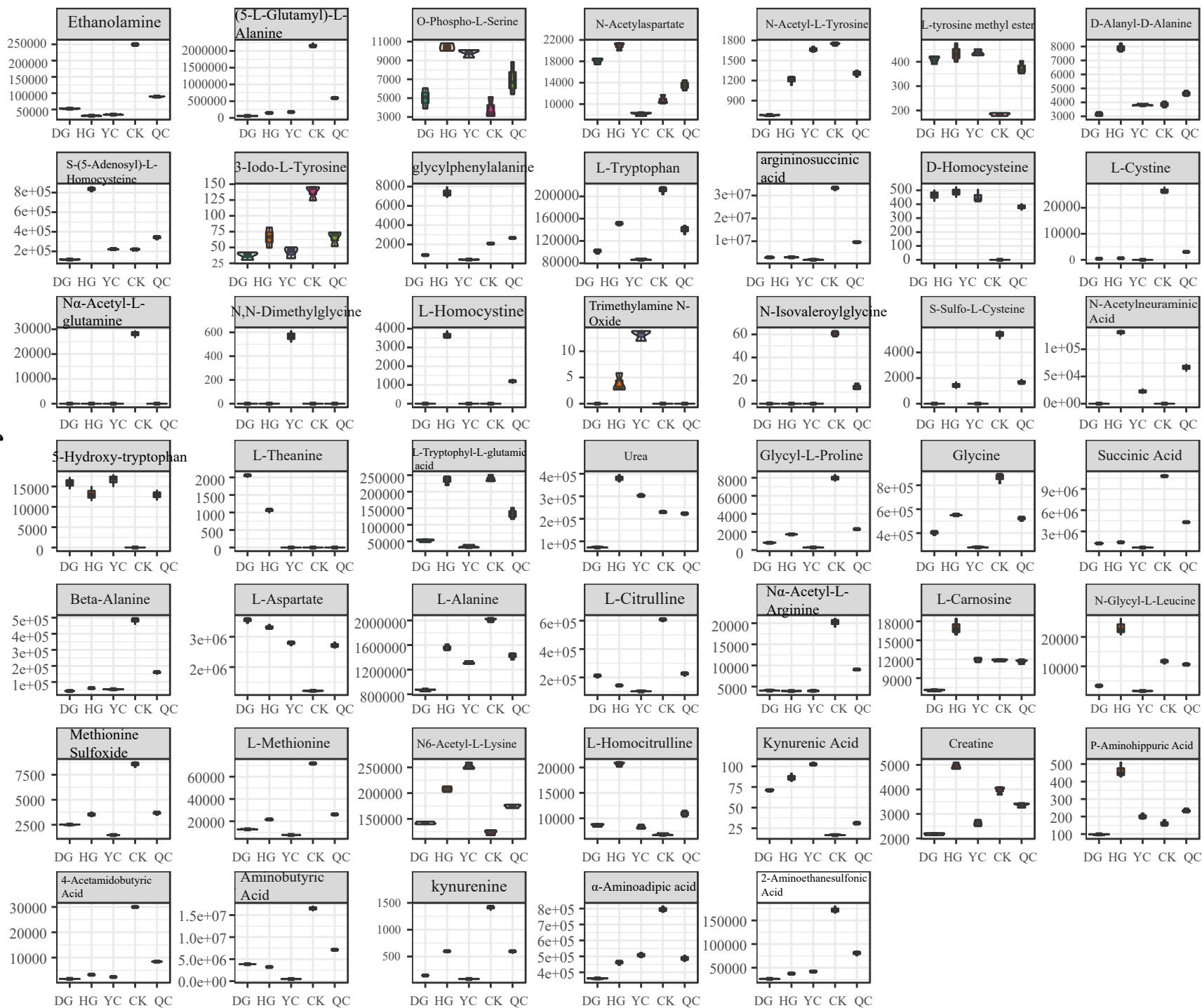

Supplement: Supplementary file 1 [file metabolites-14-00459-s001.zip › supplementary materials/FigureS3.pdf]
